# Pesticide residue survey of pollen loads collected by honeybees (*Apis mellifera*) in daily intervals at three agricultural sites in South Germany

**DOI:** 10.1371/journal.pone.0199995

**Published:** 2018-07-06

**Authors:** Franziska Böhme, Gabriela Bischoff, Claus P. W. Zebitz, Peter Rosenkranz, Klaus Wallner

**Affiliations:** 1 University of Hohenheim, Apicultural State Institute, Stuttgart, Germany; 2 Julius Kühn-Institute, Institute for Bee Protection, Berlin, Germany; 3 University of Hohenheim, Institute of Phytomedicine, Applied Entomology, Stuttgart, Germany; Institut Sophia Agrobiotech, FRANCE

## Abstract

In agricultural landscapes honeybees and other pollinators are exposed to pesticides, often surveyed by residue analysis of bee bread. However, bee bread is a mixture of pollen pellets of different plants collected over a longer time period. Therefore, pesticide content in the hive varies with plant species and time of pollen collection. Hence, the analysis of bee bread is an approximate approach to gain information on detailed pesticide exposure during the agronomic active season. As high-resolution data is missing, we carried out a pesticide residue survey over five years (2012–2016) of daily collected pollen pellets at three agricultural distinct sites in southern Germany. 281 single day pollen samples were selected and subjected to a multi-pesticide residue analysis. Pesticide contaminations of pollen differed between the sites. Intensive pesticide exposure can be seen by high pesticide concentrations as well as a high amount of different pesticides detected. During the five years of observation 73 different pesticides were found, of which 84% are characterized as non-harmful to honeybees. To estimate pesticide risks for honeybees, the pollen hazard quotient (PHQ) was calculated. Even though pesticides were detected in sublethal concentrations, we found substances not supposed to be exposed to honey bees, indicating the necessity for further improvement of seed treatments and increasing awareness of flowering shrubs, field margins and pesticide drift. Additionally, an in-depth analysis of nine pollen samples, divided into sub-fractions dominated by single plant species, revealed even higher concentrations in single crops for some pesticides. We give precise residue data of 1,657 single pesticide detections, which should be used for realistic laboratory and field tests.

## Introduction

Integrated pest management, including chemical crop protection, is an approach by farmers to ensure crop yield [1]. Actual overall yield losses due to weeds, pests and diseases range between 26 and 40% but may potentially reach 50 and 80%, depending on crop, cultivar, and region [2]. Facing a growing world population, it is crucial to guarantee continuous high yields on the available arable land but avoiding negative impacts on the environment at the same time [3]. The latter includes the protection of the farmland and the safety of the produced crops for the consumer. However, one of the biggest challenges for the use of pesticides is the protection of pollinators. For many crops, high yield is not only influenced by crop protection, production site, agricultural practice or cultivar but also by optimized pollination services [1,2,4,5]. Pollination is one of the most important services provided by honeybees (*Apis mellifera* L.), the most abundant pollinators in intensively managed agroecosystems due to a loss of wild pollinators [4,6–8].

However, pesticides not only impact the target organism, they can contaminate air, soil and surface, can drift to non-target plants or water, and can leach or run off the soil into surface water and the aquifer during direct application or indirectly by seed coating [9,10]. Stable or systemic substances can also accumulate in the soils and sediments and may be taken up by plants and transported with the vascular system [9].

Hence, there are multiple routes of exposure to pesticides for honeybees or other pollinators: direct contamination during spraying into the blossom [11], by dust deposit abraded from treated seeds during sowing [12,13], by contaminated water puddles [14], by uptake of volatilized pesticides [15], or indirectly by collection of nectar, pollen, and guttation droplets contaminated by (systemic) pesticides [16–18] of crops and even wildflowers [9,19]. Consequently, residues of pesticides can be found in bee matrices like pollen pellets, bee bread, honey, wax or royal jelly [20–24].

Residue analysis of bee bread is a common tool in monitoring studies as bee bread is accessed easily and can be stored in sufficient sampling quantities [23–28]. However, bee bread usually represents a mixture of hundreds of pollen loads, contaminated by pesticides or not, from different plants. This results in pollen pellets of varying pesticide concentration and composition within the sample. Therefore, pesticide analysis of bee bread is an approximate approach to estimate the real exposure to pesticides. A more profound view of pesticide exposure via pollen is offered, when pollen loads of returning foragers at the hive entrance are collected and analyzed [16,26,29–34]. However, in some of these studies only few samples are drawn during the season, or only few crops or some pesticide applications are monitored. Additionally, samples were pooled sometimes to receive enough analyzing material, all resulting in no detailed data to show the real pesticide exposure during the agronomic active season.

Even though sometimes high pesticide concentrations were measured, most of the detected pesticide concentrations were found in sublethal levels considering LD_50oral_ values and maximum pollen consumption rates [35]. However, it is known that even sublethal concentrations of pesticides to invertebrates would lead to effects, such as impairment of cognitive competencies, changing social interactions or side-effects on growth, development or gene expression in honeybees [36–43].

Knowing, that even small amounts of pesticides could have effects on honeybees and that there is a lack of studies with precise residue data, our main target was to reveal the exact pesticide content of pollen loads collected daily by foragers during the agronomic active season before storage inside the hive. For this purpose, we had chosen apiaries situated in different agricultural landscapes in South Germany and collected pollen pellets with pollen traps for five years. A representative number of nearly 300 pollen pellet samples, each representing a single day, were analyzed for nearly 300 active ingredients currently used in agricultural practice. The resulting high-resolution residue data is now available. Furthermore, to get a better understanding of the hazard posed to honeybees by the measured pesticide concentrations, we calculated the pollen hazard quotient (PHQ) following the method by Stoner and Eitzer [31]. In addition, we conducted in depth analyses from nine analyzed samples to identify key plant species within the pollen loads to show plant specific pesticide contamination.

## Material and methods

No permits were needed. The beekeepers were voluntarily taken part in this study ontheir personell ground. Due to privacy protection of the beekeepers the exact coordinates of the apiaries will not be given. No endangered species were involved in this study.

### Collection sites: “Meadow”, “grain” and “fruit”

In close cooperation with the respective beekeepers in the State of Baden-Württemberg, representative apiaries for agricultural used landscapes in regions with temperate climate were chosen. Within these areas the voluntarily participating beekeepers had their private apiaries. As honeybees have a vast flight range of a several kilometers radius around the hive, it was not possible to get any information on details of the respective crop protection strategies and intensity by the numerous farmers cultivating the fields in the vicinity of the apiaries. Furthermore, the crop protection intensity usually varies from year to year due to the prevailing weather conditions and pressure by damaging factors. The sites are at least 50 km apart from each other. The “meadow” site (323 m a.s.l.; meters above sea level) is located in the north-east of the town Göppingen. The area around the apiary is characterized by about 60% permanent grassland, i.e. pasture, meadow, orchard meadows and traditional extensive orchards. “Grain” site (569 m a.s.l.) is situated in the east of the village Ertingen, at the edge of a forest. It is characterized by more intensive agriculture with high percentages of small grains (wheat, barley, oat, etc.), grain and silage maize, winter oilseed rape and meadows. “Fruit” site is in the south of Heilbronn (157 m a.s.l.). 40% small grains and maize, as well as 30% permanent crops such as vine, pome, stone and soft fruits, characterize this site. Additional information for each site is given in Table A-C in S1 Tables and in Table A-C in S2 Tables.

### Pollen collection

Pollen traps were set up from spring (March) until late summer (August), depending on the site, at the entrance of one honeybee colony at each of the three locations in Southern Germany. Pollen collection started 2012 and ended 2016 for the “meadow” and “grain” sites. The beekeeper of the “fruit” site collected pollen three years from 2012 to 2014. The place of the colonies remained the same during the whole experimental period (= stationary beekeeping). Bee colonies were maintained according to good apicultural practice. Pollen pellets were collected daily or every second day by each beekeeper and stored separately in labeled plastic bags at -20°C. Pollen pellets were shipped frozen to the Apicultural State Institute where they were stored under -20°C until chemical analysis.

Due to financial limitations 9–39 samples for each site and sampling period were chosen and sent to the laboratories for pesticide residue analysis. Depending on the quality (uncontaminated with debris and not slushy) and quantity (more than 5 g pollen per day), samples were selected randomly to cover the entire sampling period of beekeeping. This resulted in varying numbers of samples for each month, year and site, with at least one sample each week. Yet, samples have not been pooled and results represent the pesticide contamination of a single sampling day from one apiary.

### Pesticide residue analysis

Residue analysis of a total of 281 samples (over the three sites and three to five years), each > 5 grams, have been performed at two laboratories. The agricultural analytic and research institute in Speyer (LUFA Speyer) performs a multi-residue QuEChERS method following Anastassiades et al. [44] (§ 64 LFGB, BVL L 00.00-115/1:2015–03) following an analysis by GC-MS(/MS) and LC-MS/MS. The Julius Kühn-Institute (JKI) uses a multi-residue method as described by Böhme et al. [45]. Both laboratories were looking for 282 different substances (pesticides and some metabolites) currently used in agricultural practice.

The following samples were analyzed by (i) LUFA Speyer: “grain”: 2012–2016, “meadow”: 2015 and 2016, in depth analysis of sub-fractions of predominantly one plant species (see below “Fractions of pollen samples”); by (ii) JKI: “meadow”: 2012–2014, “fruit”: 2012–2014.

In order to compare all samples from both laboratories, the highest limit of detection (LOD) and limit of quantification (LOQ) for each substance from the analysis by LUFA Speyer had to be chosen and the results obtained from the laboratory of the JKI were considered accordingly. Values or substances below this LOQ were not considered for the comparison.

### Residue evaluation

To estimate the hazard to bees emanating from contaminated pollen loads, the pollen hazard quotient (PHQ) was calculated following Stoner and Eitzer [31]. This method was chosen as it provides a simple and comprehensive way to calculate the risk based on LD_50_-values easily available in the internet. The concentration of each pesticide found in a sample (μg/kg) was divided by the LD_50_ (honeybee oral; μg/bee) for the respective substance. LD_50_ values were obtained from the University of Hertfordshire pesticides properties database [46], the US EPA ecotoxicology database [47] or the Agritox database of the French government [48]. PHQ calculations for thymol based on LD_50_ concentrations (honeybee contact) by Dahlgren et al. [49]. For metabolites, the respective LD_50_ value of the parent pesticide was used for calculations. Total PHQ per sample (= day; tPHQ_day_) was calculated as the sum of all PHQs of the pesticides in the respective sample.

PHQ of > 50 are considered “relevant”. Assuming a daily pollen consumption of 9.5 mg by a nurse bee [24,31,35], a PHQ of 50 would correspond to 0.05% of the LD_50_ consumed in one day (resulting in 0.5% of the LD_50_ in an average 10-day nursing period) [31].

#### Statistical analysis

PHQ and tPHQ_day_ values were analyzed using the computer software JMP ^®^ 11.1.1 (SAS Institute Inc., Cary, NC, USA) as follows: (i) analysis of variance using a generalized linear model (GLM procedure) for effects by site, year, and month, and (ii) by one-way ANOVA followed by pairwise Student-t test between sites and years.

Each active substance is assigned the respective mode of action class following the classification of the FRAC [50], IRAC [51] and HRAC [52] (Table 1). Since several active substances share the same mode of action, the concentration of each substance at a single day were summed. Grouped pesticides were then analyzed statistically as described above. In the same way total insecticides/day and total fungicides/day were analyzed.

**Table 1:** Summary of 73 pesticides detected in corbicular pollen loads of honeybees at three distinct agricultural production sites in South Germany during 2012–2016.

Substance	Class, systemicity a)	Group b)	Code b)	Likelihood of appearance in pollen c) [53]	Appearance in pollen not expected d) [53]	LOD (μg/kg) e)	LOQ (μg/kg) e)	Total detections f)	Highest detected concentration (μg/kg)	LD50oral g)	MRL for apicultural products (μg/kg) h) [54]	PHQmax
Acetamiprid	I, s	Neonicotinoids	4A	x		2	5	21	42.7	14.53	50	2.94
Azoxystrobin	F, s	QoI	11	x		2	5	40	560.5	25	50	22.42
Benthiavalicarb isopropyl	F	CAA	40	x		2	5	2	8.9	100	50	0.09
Boscalid	F	SDHI	7	x		2	5	77	1,496.4	100	50	14.96
Chlorantraniliprole	I	Diamides	28	x		5	10	1	18.1	104.1	50	0.17
Clothianidin	I, s	Neonicotinoids	4A		x	2	5	3	2.4	0.004	50	600
Coumaphos	V	Organophosphates	1B		x	2	5	1	3.5	4.61	10	0.76
Cyflufenamid	F	Phenylacetamide	U6	x		2	5	8	20.1	100	50	0.20
Cyproconazole	F, s	DMI (SBI: Class I)	3	x		2	5	1	27.7	1,000	50	0.03
Cyprodinil	F, s	AP	9	x		2	5	40	1,282.6	112.5	50	0.22
Difenoconazole	F, s	DMI (SBI: Class I)	3	x		2	5	11	147.7	177	50	0.83
Dimethoate	I, s	Organophosphates	1B		x	2	5	2	19.7	0.12	-i)	164.41
Dimethenamid-P	H	Chloroacetamides	K3	x		2	5	8	11.8	118.4	50	0.10
Dimethomorph	F, s	CAA	40	x		2	5	15	2,678.4	32.4	50	82.67
Dimoxystrobin	F	QoI	11	x		2	5	42	576.2	79.4	50	7.26
Epoxiconazole	F	DMI (SBI: Class I)	3	x		2	5	19	170.4	83	50	2.05
Etofenprox	I	Pyrethroids	3A	x		2	5	9	7.8	0.27	50	28.89
Famoxadone	F	QoI	11	x		2	5	1	2.8	1,000	50	0.003
Fenhexamid	F	SBI: Class III	17	x		2	5	18	7,177.7	102.07	50	70.32
Fenoxycarb	I	Fenoxycarb	7B		x	2	5	1	6.3	204	50	0.03
Fenpropimorph	F, s	SBI: Class II	5	x		2	5	12	17.7	95.6	50	0.19
Fluazifop-butyl	H, s	FOP	A	x		5	15	45	6,831.3	63	50	108.43
Fludioxonil	F	PP	12	x		5	15	32	1,085.1	100	50	10.85
Fluopicolide	F	Benzamides	43	x		2	5	8	23.15	241	50	0.21
Flonicamid	I, s	Flonicamid	29	x		2	5	1	7.3	60.5	50	0.12
Fluopyram	F	SDHI	7	x		2	5	25	134.3	102.3	50	1.31
Fluroxypyr-methyl	H	Pyridine-carboxylic acids	0	x		5	10	2	242.5	100	50	2.42
Fluoxastrobin	F, s	QoI	11	x		2	5	1	2	843	50	0.002
Flusilazole	F, s	DMI (SBI: Class I)	3	x		2	5	2	115.6	33.8	50	3.42
Fuberidazole	F, s	MBC	1		x	2	5	4	12.9	187.2	50	0.07
Gamma-, Lambda-Cyhalothrin	I	Pyrethroids	3A	x		2	5	2	2.5	2.56	50	0.98
Imidacloprid	I, s	Neonicotinoids	4A		x	2	5	1	2.1	0.0037	50	567.57
Indoxacarb	I	Oxadiazines	22A	x		2	5	5	20	0.26	50	76.92
Iprovalicarb	F, s	CAA	40	x		2	5	11	974.7	199	50	4.90
Isoproturon	H, s	Urea	C2	x		2	5	15	23.3	195	50	0.12
Kresoxim-methyl	F	QoI	11	x		2	5	10	106.3	110	50	0.97
Mandipropamid	F	CAA	40	x		2	5	8	101.8	200	50	0.51
MCPA	H	Phenoxy-carboxylic-acids	0	x		5	15	1	667	200	-i)	3.34
Metamitron	H, s	Triazines	C1	x		2	5	1	6.6	97.2	-i)	0.07
Metalaxyl-M	F, s	PA	4	x		2	5	9	17.6	97.3	50	0.18
Metconazole	F, s	DMI (SBI: Class I)	3	x		2	5	20	94.4	85	50	1.11
Metolachlor	H	Chloroacetamides	K3	x		2	5	5	6.2	110	50	0.06
Methiocarb	I	Carbamates	1A		x	2	5	29	47.7	0.47	50	101.54
Methiocarb-sulfoxide	I	Carbamates	1A		x	2	5	1	2.5	0.47	50	5.33
Methoxyfenozide	I	Diacylhydrazines	18	x		2	5	4	14.4	100	50	0.14
Metrafenone	F	Aryl-phenylketones	U 08	x		2	5	35	368.5	114	50	3.23
Myclobutanil	F, s	DMI (SBI: Class I)	3	x		2	5	16	136.8	33.9	50	4.04
Nicotine	I, s	Nicotine	4B		x	2	5	1	3	0.08	-i)	37.5
Penconazole	F, s	DMI (SBI: Class I)	3	x		2	5	18	35	112	50	0.31
Pencycuron	F	Phenylureas	20		x	2	5	2	4.5	98.5	-i)	0.04
Pendimethalin	H	Dinitroanilines	K1	x		3	10	33	43.4	101.2	50	0.43
Picaridin	IR	Piperidines	-		x	2	5	37	412	-j) [55,56]	-j) [55,56]	-j)[55,56]
Picoxystrobin	F	QoI	11	x		2	5	1	4.2	200	50	0.02
Pirimicarb	I, s	Carbamates	1A	x		2	5	2	14.7	4	50	3.68
Pirimicarb-desmethyl	I	Carbamates	1A	x		2	5	1	4.1	4	50	1.03
Propiconazole	F, s	DMI (SBI: Class I)	3	x		2	5	2	6.5	100	50	0.07
Propamocarb	F, s	Carbamates	28	x		2	5	2	8	84	50	0.10
Proquinazid	F	Azanaphthalenes	13	x		5	10	13	110.2	125	50	0.88
Prosulfocarb	H	Thiocarbamates	N	x		2	5	38	24.2	103.4	50	0.23
Prothioconazole-desthio	F, s	DMI (SBI: Class I)	3	x		2	5	100	78.6	71	50	1.11
Pyraclostrobin	F	QoI	11	x		2	5	15	124	72.1	50	1.72
Pyrimethanil	F	AP	9	x		2	5	1	2.6	100	50	0.03
Quinoxyfen	F, s	Azanaphthalenes	13	x		5	10	10	261.4	1,000	50	0.26
Spiroxamine	F, s	SBI: Class II	5	x		2	5	9	132.4	100	50	1.32
Tau-Fluvalinate	I	Pyrethroids	3A	x		2	5	2	10	12.6	50	0.80
Tebuconazole	F, s	DMI (SBI: Class I)	3	x		2	5	81	484.5	83.05	50	5.83
Terbuthylazine	H	Triazines	C1	x		2	5	11	5.4	22.6	-i)	0.24
Tebufenozide	I	Diacylhydrazines	18	x		2	5	5	24.8	100	50	0.25
Triadimenol	F	DMI (SBI: Class I)	3	x		3	10	23	80.8	224.8	100	0.36
Thiacloprid	I, s	Neonicotinoids	4A	x		2	5	145	470.4	17.32	200	27.16
Thymol	V	Thymus vulgaris essential oil	-		x	2	5	1	8	56.6	No MRL required	0.14
Trifloxystrobin	F	QoI	11	x		2	5	27	218.3	200	50	1.09
Zoxamide	F	Benzamides	22	x		2	5	1	6.6	100	50	0.07

^a)^ F = fungicide, H = herbicide, I = insecticide, IR = insect repellent V = varroacide, s = systemic

^b)^For fungicides: FRAC Code List, for herbicides: HRAC, for insecticides: IRAC Classification Scheme; AP = Anilino-Pyrimidines; CAA = Carboxylic Acid Amides; DMI = DeMethylation Inhibitors; FOP = Aryloxyphenoxy-propionate, MBC = Methyl Benzimidazole Carbamates; PA = PhenylAmides; PP = PhenylPyrroles; QoI = Quinone outside Inhibitors; SBI = sterol biosynthesis inhibitors; SDHI = Succinate dehydrogenase inhibitors

^c)^ At least one authorized plant protection product containing this active ingredient (a.i.) is classified B2 or B4. B2 = classified as hazardous to bees, except when applied after the end of the daily bee flight, B4 = classified as non-hazardous to bees

^d)^ At least one authorized plant protection product containing this active ingredient is classified B1 or B3. B1 = classified as hazardous to bees, B3 = Due to the manner in which authorization governs application of the product, bees are not endangered. Coumaphos, nicotine, picaridin and thymol are not authorized as plant protection products.

^e)^ LOD and LOQ from LUFA Speyer had to be chosen for better comparison of all sites.

^f)^ Total detections after LOD and LOQ were chosen from LUFA Speyer.

^g)^ LD_50_ (honeybee oral): PPDB (2017), US EPA (2017), Agritox database (2017), Dahlgren et al. (2012)

^h)^ MRL = maximum residue limit for products of animal origin, EU pesticides database

^i)^ No MRL data for these substances are listed in the EU pesticides database

^j)^ No data regarding ecotoxicological analysis concerning bees are available; no tolerance levels for picaridin in food are given as residues are not expected due to its use as topical insect repellent

### Fractions of pollen samples

After receiving the results of the residue analysis, nine samples were chosen for in depth analysis to show the contribution of plant specific pesticide residues to the pesticide contamination of the whole sample. The pollen pellets remaining after taking five grams for residue analysis (correspond here to composite sample), were separated according to their color (correspond here to fraction). A subsample of the color fraction was ground, dissolved in water with a droplet of a tensioactive agent (soap), spread on a microscopic slide, left to dry and embedded with Kaiser’s glycerol gelatin and a cover glass. Light microscopic palynological analyses were performed at the honey laboratory at the Apicultural State Institute before fractions were sent to LUFA Speyer for pesticide residue analysis.

## Results

### Pesticide residues

**Fig 1:**
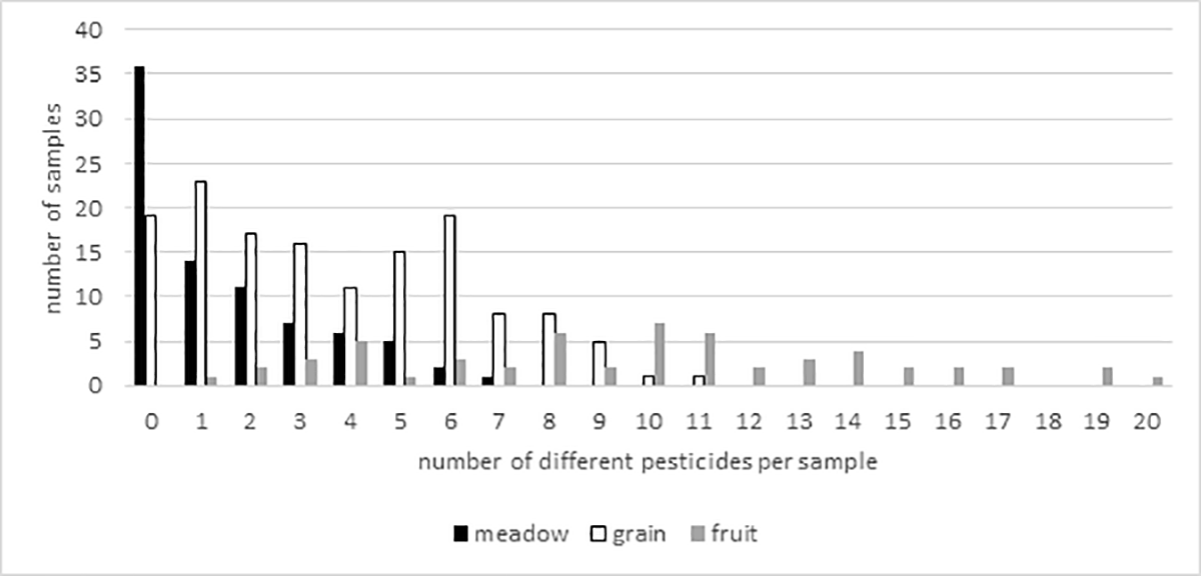
Frequency of contaminated pollen trap samples (n = 281) separated for each site overall years. “Meadow” and “grain” were sampled from 2012–2016, “fruit” was sampled 2012–2014.

Altogether, 281 honeybee corbicular pollen load samples from three locations were analyzed in a multi-residue screening. One fifth of all samples were found uncontaminated. In total 73 different pesticides, comprising herbicides, fungicides and insecticides (including varroacides and one insect repellent), and some of their metabolites were detected, of which 31 substances have systemic properties (Table 1). At “fruit” 58 different substances were detected in only three sampling years, followed by “grain” with 37 and “meadow” with 24 different substances in five sampling years. At all sites and years fungicides account for more than 50% of the detected substances (Table 2). Generally, the least intensive chemical crop protection activity was identified at the “meadow” site, considering number of detected pesticides, number of pesticide detections and maximum pesticide concentrations. Most of the samples free of pesticide contamination were collected at “meadow” (43.9%) with a mean pesticide load of 2.0 pesticides per sample, followed by “grain” (13.3%) and 3.8 pesticides per samples (Fig 1). All pollen samples collected at the “fruit” site contained, on average 9.8 pesticides per sample. The lowest maximum concentrations were measured at “meadow”, followed by “grain” and further exceeded by “fruit” with 294.6, 1,496.4 and 7,177.7 μg/kg, respectively. (Table A-C in S3 Tables and S4 Tables).

**Table 2:** Summary of main pesticide residue results of honeybee collected pollen loads for each site and year.

	Meadow^a)^					grain					fruit ^a)^		
Years	2012	2013	2014	2015	2016	2012	2013	2014	2015	2016	2012	2013	2014
Sampling dates	02.04.-07.06.	01.05.-10.08.	12.04.-05.05.	21.04.-10.07.	05.05.-30.07.	26.03.-30.07.	18.04.-28.07.	01.04.-06.07.	21.04.-21.07.	19.04.-21.07.	01.04.-25.06.	08/09.05.-01/02.08.	20/21.04.-7/8.06.
Nr. analyzed samples (% positive samples)	16 (50)	22 (40.9)	9 (100)	19 (78.9)	16 (31.3)	39 (84.6)	30 (70)	20 (80)	27 (100)	27 (100)	24 (100)	20 (100)	12 (100)
Nr. different pesticides (F/I/H)^b)^	7 (6/1/0)	9 (7/1/1)	7 (5/1/1)	8 (6/2/0)	9 (4/2/3)	17 (9/4/4)	14 (9/2/3)	23 (11/5/7)	25 (14/5/6)	18 (12/3/3)	44 (27/12/5)	40 (27/10/3)	34 (26/4/4)
Min/max amount of pesticides per sample	0/2	0/7	2/5	0/6	0/5	0/8	0/7	0/11	1/10	1/9	1/18	4/20	4/16
Range of concentrations (min-max; μg/kg)	2.3–13.2	2.0–294.6	2.0–11.1	2.0–105.3	2.1–19.2	2.0–364.3	2.2–191.3	2.1–871.6	2.1–315.6	2.0–1,496.4	2.0–841.7	2.0–7,177.7	2.0–6,831.3
min-max tPHQ_day_ (n) = times ≥ 50	0.00–0.78	0.00–46.06	0.19–6.85	0.00–9.87	0.00–0.42	0.00–126.50 (2)	0.00–75.78 (2)	0.00–107.51 (1)	0.00–221.99 (1)	0.00–35.67	0.04–534.81 (1 ≥ 500)	0.20–615.48 (3, 3 ≥ 500)	0.68–125.89 (6)
Month with most pesticide detections (% of annual detections)	May (63.6)	May (96.8)	April (84)	May (45.5)	May (100)	May (46.4)	May (65.1)	April (48.4)	May (51.2)	May (45.8)	June (41.5)	June (39.6)	May (56.9)
Substances with the highest concentrations: max. concentration (μg/kg)	trifloxystrobin: 13.2, thiacloprid: 12.3, pencycuron: 4.5	thiacloprid: 294.6, dimoxystrobin: 161.6, boscalid: 75.9	fluazifop 11.1, metconazol: 8.9, dimoxystrobin: 7.4	thiacloprid: 105.3, dimoxystrobin: 93.8, boscalid: 62.6	fluopyram: 19.2, picaridin: 18.7, prothioconazole-desthio: 8.7	thiacloprid: 364.3, triadimenol: 72.7, tebuconazole: 50.0	thiacloprid: 191.3, fluopyram: 134.3, triadimenol: 80.8	fluazifop: 871.6, thiacloprid: 111.9, fluopyram: 59.7	picaridin: 315.6, fluroxypyr-methyl: 242.5, thiacloprid: 103.4	boscalid: 1,496.4, dimoxystrobin: 576.2, picaridin: 412.0	dimethomorph: 841.7, MCPA: 667,.0 thiacloprid: 470.4	fenhexamid: 7,177.7, dimethomorph: 2,678.4, iprovalicarb: 974.7	fluazifop: 6,831.3, cyprodinil: 1,282.6, fludioxonil: 1,085.1
Most frequent pesticides; (n) = times detected per year	thiacloprid (5)	thiacloprid/ fluazifop (each 7), boscalid (4)	metconazol (8), fluazifop (6), thiacloprid (5)	thiacloprid/ cyprodinil (each 9), boscalid (7), prothioconazole-desthio(6)	prothioconazole-desthio (4), fluopyram/ prosulfocarb (each 2)	thiacloprid (25), tebuconazole (17), prothioconazole-desthio (15)	prothioconazole-desthio (16), thiacloprid (14), dimoxystrobin (11)	thiacloprid (13), prothioconazole-desthio (12), fluazifop (11)	prothiconazole-desthio (19), picaridin/ thiacloprid (each 13), pendimethalin/ tebuconazole (each 10)	picaridin (23), prothioconazole-desthio (13), tebuconazole (9)	thiacloprid (21), Boscalid (16), tebuconazole (12)	cyprodinil/ fludioxonil (each 17), thiacloprid (15), boscalid (13)	thiacloprid (10), prosulfocarb (9), fluazifop (8)

^a)^ Comparison of sites based on the LOD/LOQ as obtained by LUFA Speyer.

^**b)**^ F = fungicides, I = insecticides, H = herbicides

In all years at “meadow” and “grain”, the months April and May revealed the majority of contaminated samples as well as most pesticide detections, whereas most of the uncontaminated or less contaminated samples were found in the summer months June and July. At “fruit” site all months showed high pesticide frequency, with high contamination also in June or July (Table 2, Fig 1).

Due to the classification of the respective plant protection products according to German authorization, 83.6% of the measured substances are likely to appear in pollen, due to permitted application into the flower. Twelve of the substances found are not supposed to appear in pollen. Eight substances (and some of their metabolites) are classified harmful to adult bees or bee brood (i.e. methiocarb, clothianidin, imidacloprid, dimethoate, fenoxycarb, pirimicarb, indoxacarb, nicotine) (Table 1).

### Pollen hazard quotient

Pollen hazard quotients (PHQ) are calculated based on LD_50_ values and therefore represent the toxicity of substances. Hence, very toxic substances yield in high PHQ values. The PHQ values ranged between 0.002 and 600 within all observation years, sites and pesticides. At “meadow” site the lowest PHQ were calculated and did not exceed relevant thresholds (max. PHQ 25.56).

The “relevant threshold of 50” [31] has been exceeded six times by the pesticides methiocarb and dimethoate at “grain” site (max. 164.41) and twelve times at “fruit” site by the pesticides dimethomorph, fenhexamid, fluazifop and indoxacarb. Clothianidin and imidacloprid exceeded at “fruit” site four times even a threshold of 500 (max. 600; Table 2) during the three years of observation.

Statistical analysis of PHQ values for some pesticide groups (DMI-fungicides, QoI-fungicides and pyrethroids) did not significantly differ between “meadow” and “grain”, yet both sites differed significantly in all groups from “fruit” site (Table 3).

**Table 3:** Honeybee risk exposure to pesticide groups as expressed by PHQ. PHQ values of single detections in the same sample of the same pesticide group were added (mean ± SEM). One-way-ANOVA, followed by a pairwise Student t-test at α = 0.05.

Pesticide groups^a)^	meadow	grain	fruit	F	d.f.	p
**DMI–fungicides**^**b)**^	0.033 ± 0.087 a	0.205 ± 0.066 a	0.905 ± 0.105 b	22.4721	2, 278	< 0.0001
**QoI–fungicides**^**b)**^	0.065 ± 0.186 a	0.171 ± 0.141 a	0.912 ± 0.225 b	4.9262	2, 278	0.0079
**non bee-toxic neonicotinoids**^**c)**^	0,597 ± 0,425 a	1,753 ± 0,322 b	4,287 ± 0,514 c	15.6119	2, 278	<0.0001
**Pyrethroids**	0.723 ± 0.378 a	0.010 ± 0.286 a	1.989 ± 0.458 b	6.7775	2, 278	0.0013

^a)^ means in a line followed by the same letter do not differ significantly.

^b)^ Abbreviations see Table 2

^c)^ Acetamiprid, thiacloprid; clothianidin and imidacloprid were detected only at “fruit” site

Considering the sum of all PHQs per sample and day, which represents the total pesticide load (tPHQ_day_), seven samples of “grain” (4.9%), and 13 samples of “fruit” (23.2%) exceed a tPHQ_day_ value of 50, of which values of four days even exceed a threshold of 500 (Table 2). In general, statistical differences were found amongst the three sites but not between years and months (GLM, Σ^2^ = 34.265133, d.f. = 2, 11, p < 0.0001). However, looking at each year, significant differences among sites were found only in 2013 and 2014, with “fruit” site being different from the other sites (2013: F = 6.6353, d.f. = 2, 69, p = 0.0023 and 2014: F = 10.8483, d.f. = 2, 38, p = 0.0002, respectively).

Taking the sum of all fungicides and insecticides into account, the factor site shows significant differences, while year and month do not differ, with the “fruit” site showing a significant heavier pesticide load compared to both other sites (GLM, Σ^2^ = 34.974217, d.f. = 2, 11, p < 0.0001 and GLM, Σ^2^ = 18.83227, d.f. = 2, 11, p < 0.0001, respectively).

### Maximum residue limit

**Fig 2:**
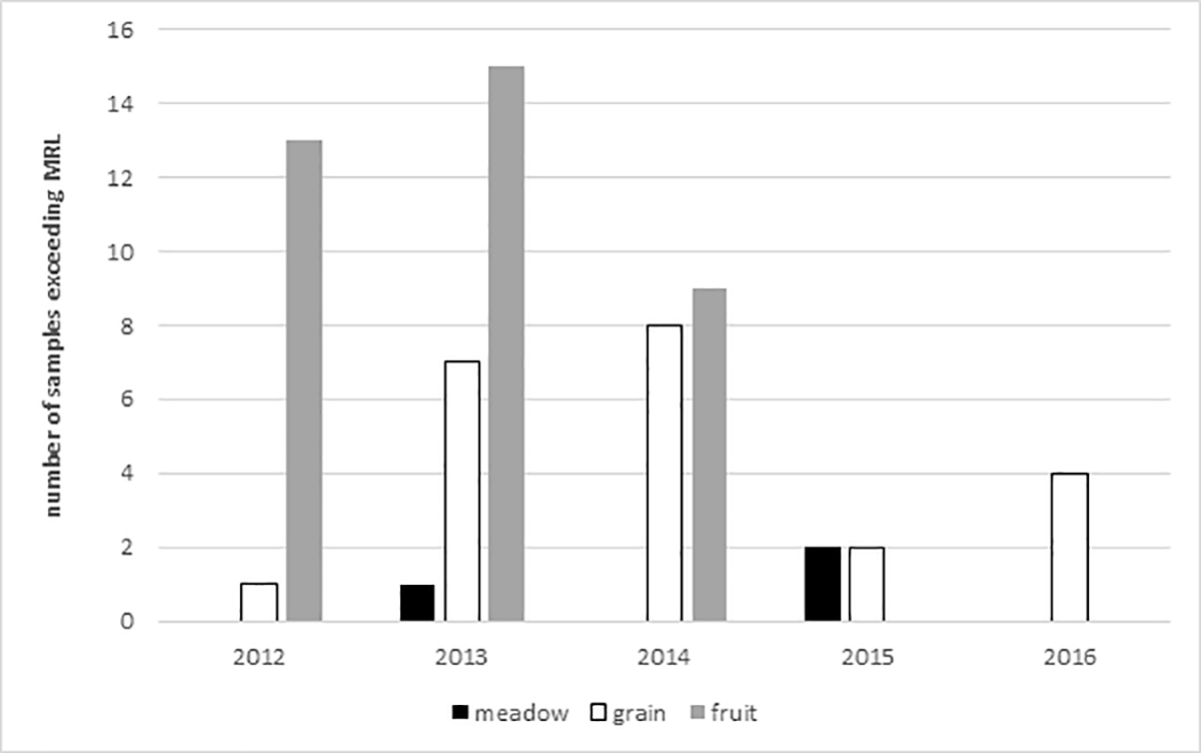
Number of pollen samples for each site and year that exceed maximum residue limits for apicultural products (MRL, μg/kg) for at least one pesticide per sample. “Meadow” and “grain” were sampled from 2012–2016, “fruit” was sampled 2012–2014.

Considering the maximum residue limits (MRL) of pesticides in apicultural products, 27 different pesticides exceeded their limits (three pesticides at “meadow”, 10 pesticides at “grain”, 25 pesticides at “fruit”). In total, up to 2, 8, or 15 samples in a single year for “meadow”, “grain”, and “fruit”, respectively, would have been classified “not marketable” (Table 1, Fig 2).

### In depth residue analysis of color-sorted pollen pellet fractions

**Fig 3:**
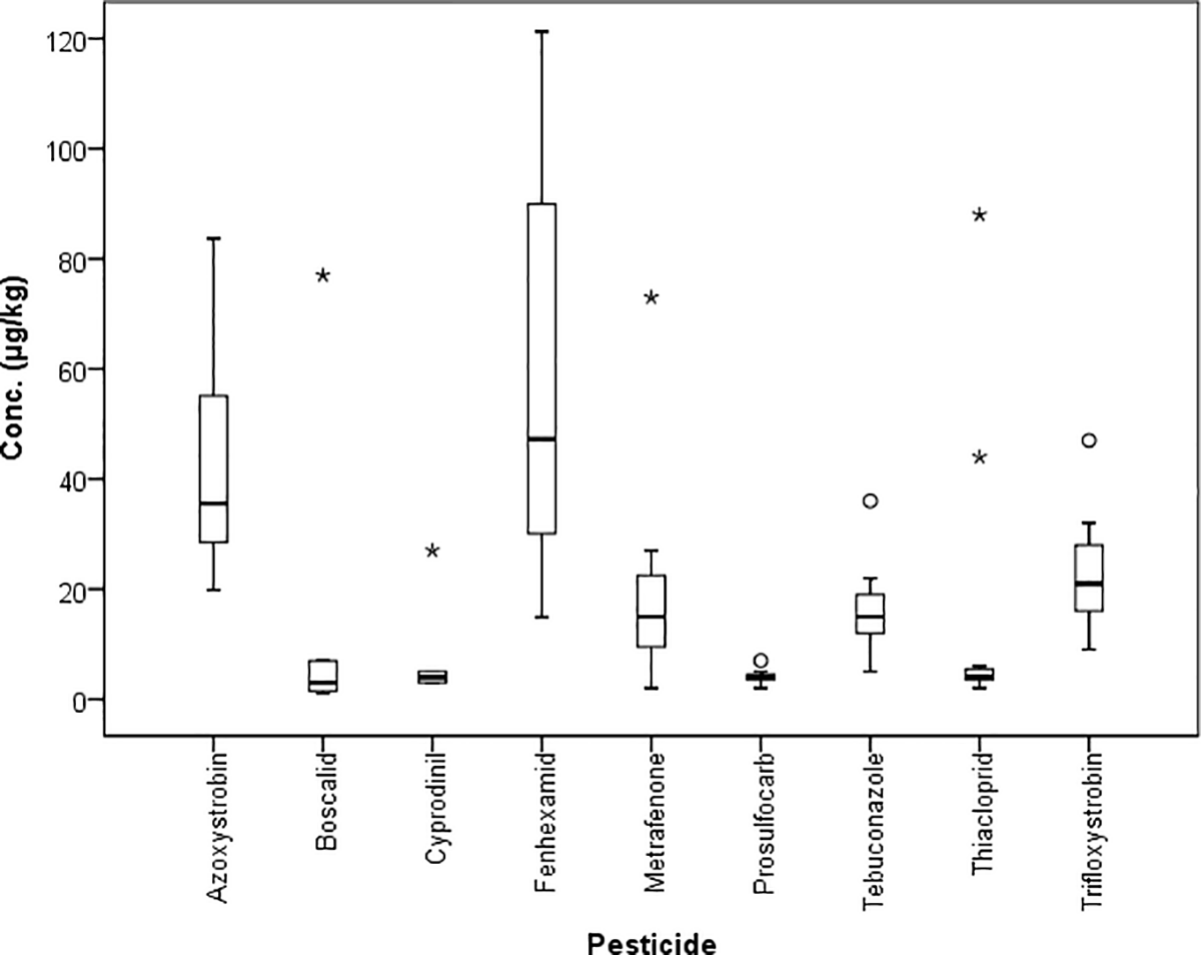
Box-and-whisker-plot of the range of concentrations (μg/kg) of pesticides being identified in at least 8 sub-fractions of the composite sample (06.06.2013) of “fruit” site. Extreme concentrations exceeding 120 μg/kg: azoxystrobin 567.8 μg/kg, boscalid 207.5 μg/kg, cyprodinil 452.3 μg/kg, fenhexamid 4,452.4 μg/kg, tebuconazole 2,589.3 μg/kg, trifloxystrobin 589.4 μg/kg. Circles and stars indicate outliers.

**Fig 4:**
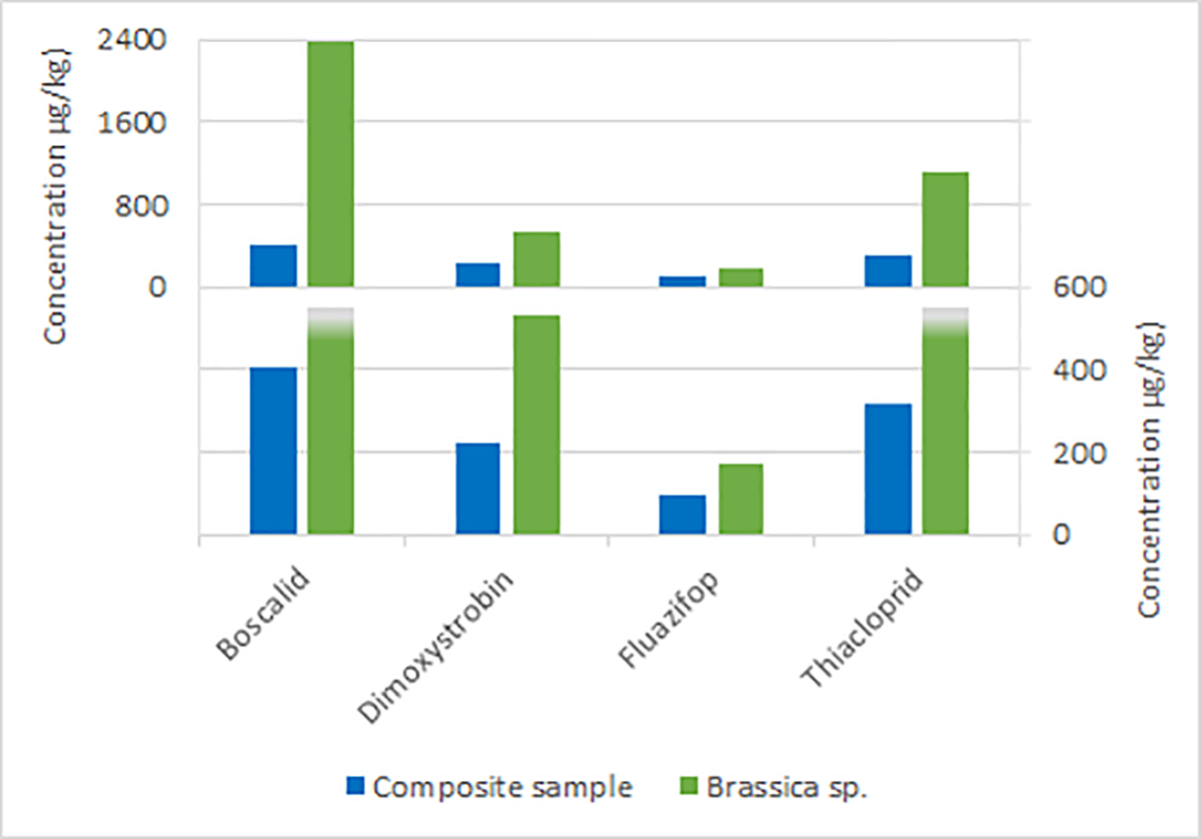
Comparison of the concentrations of the same pesticides detected in the composite sample of 03.05.2012 of “fruit” site and in the sub-fraction *Brassicae* sp. of the same sample.

**Fig 5:**
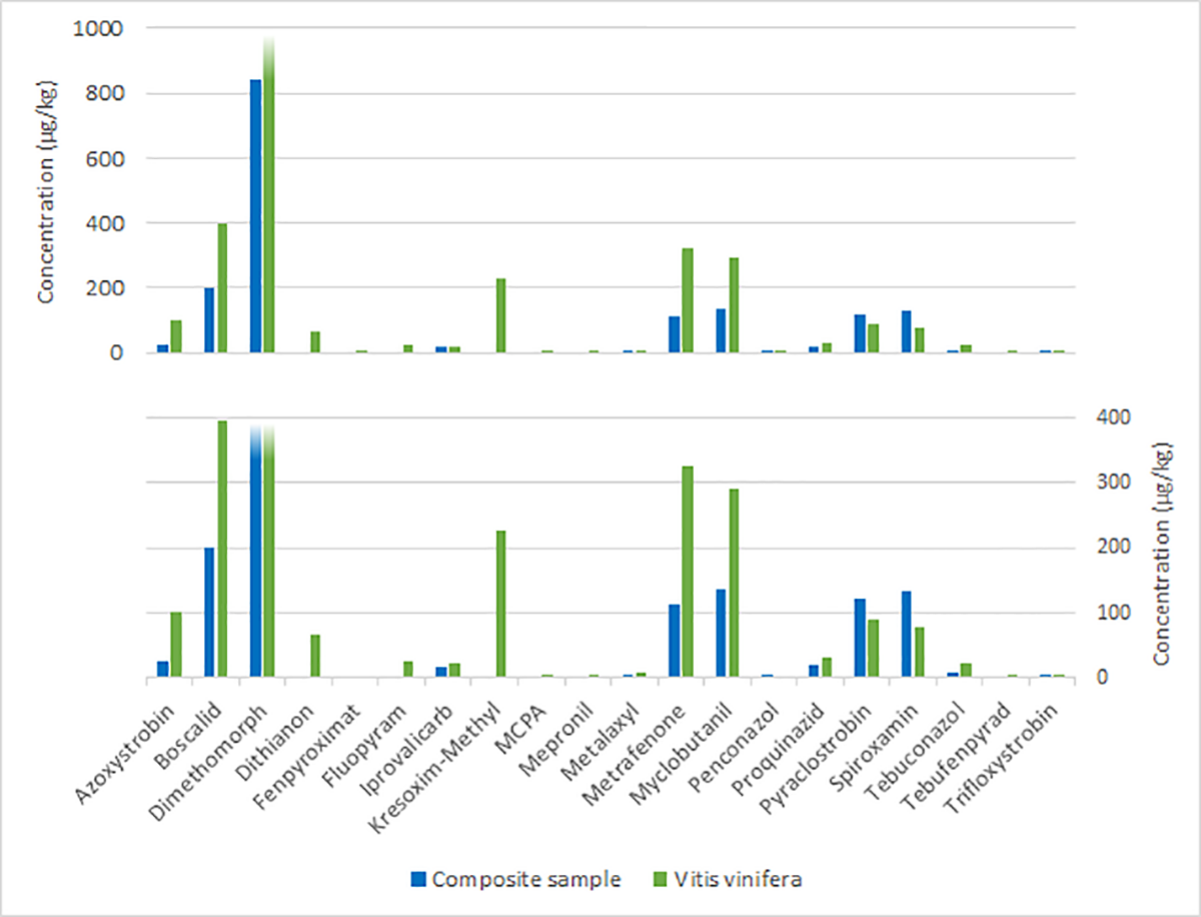
Comparison of the concentrations of the same pesticides detected in the composite sample of 14.06.2012 of “fruit” site and in the sub-fraction *V*. *vinifera* of the same sample. Dimethomorph has an extreme value with 3,747.70 μg/kg in the sub-fraction.

**Fig 6:**
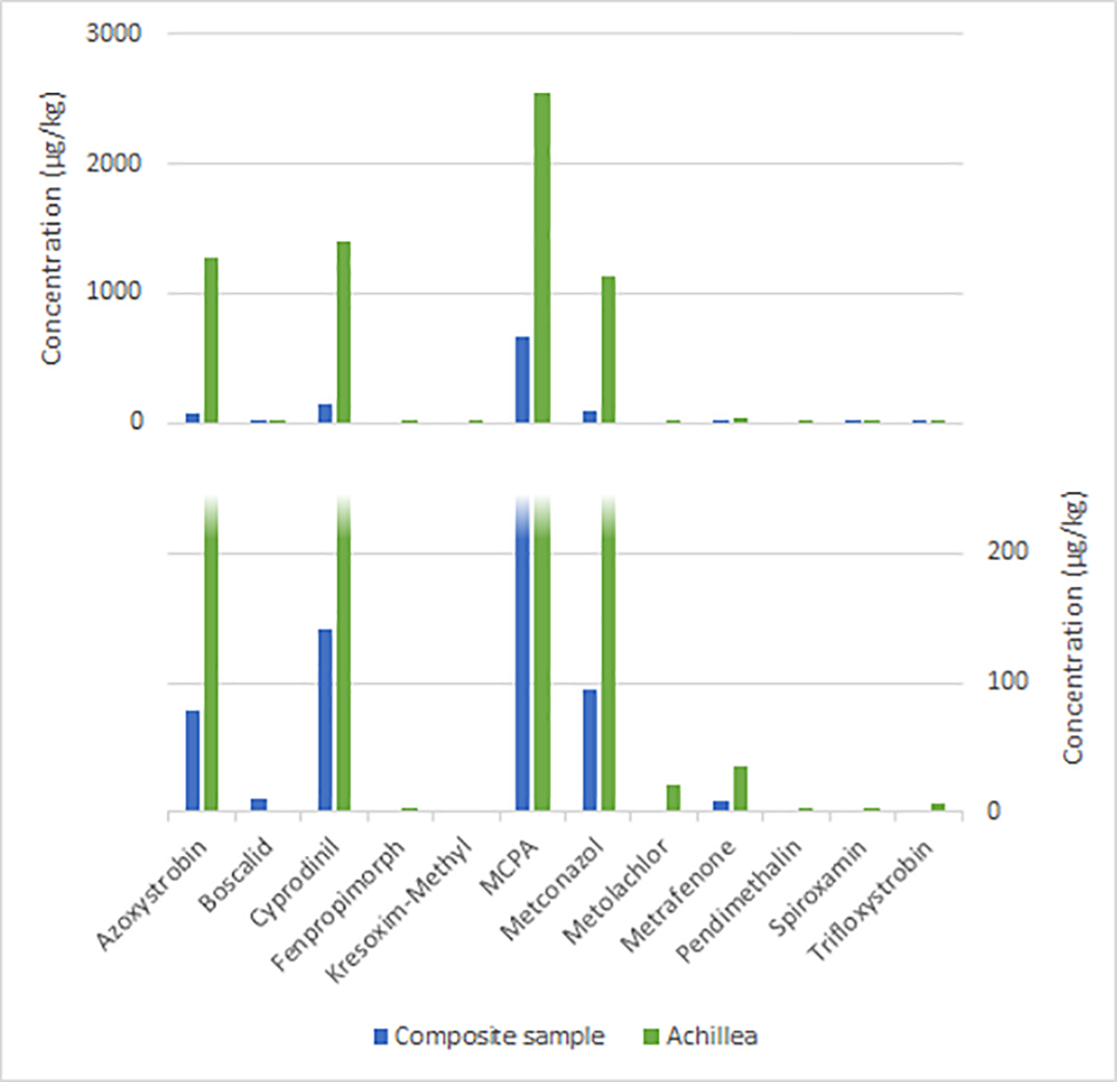
Comparison of the concentrations of the same pesticides detected in the composite sample of 28.05.2012 of “fruit” site and in the sub-fraction *Achillea* of the same sample.

In depth analysis of nine previously analyzed samples (each corresponds here to a composite sample) revealed pesticide contamination caused by predominantly one plant species. Each of the nine chosen samples was sorted according to pollen pellet color resulting in five to twelve fractions of plant species (correspond here to fraction). In many cases, active substance concentrations in the fractions exceeded the concentrations found of the same substances in the composite sample (e.g. up to 1,600 x for fenhexamid in the sample from June 14, 2012) (Table A-I in S5 Tables). Some substances were detected in low concentrations in the fractions that were not found in the composite samples before. Frequently, the same substances are detected in multiple fractions and varying widely in the range of their concentrations. The range of concentrations is shown in a box-and-whisker-plot exemplarily for one date (06.06.2013, “fruit” site), for pesticides being detected in at least eight sub-fractions (Fig 3). Furthermore, single crops, such as *Brassicae* sp. or grapevine (*Vitis vinifera* L.) show high pesticide pollution (Figs 4 and 5). Some tree or weed species, such as false acacia (*Robinia pseudoacacia* L.), maple (*Acer* L.) or *Ranunculus* L. or dandelion (*Taraxacum* F. H. Wigg.), were also contaminated, even though in lower concentrations. However, in some commonly bee pollinated weed species, such as *Achillea* L., high concentrations of pesticides could be detected (Fig 6).

## Discussion

This long-term study provides for the first time detailed information on the daily pesticide intake into honeybee colonies through pollen foragers during the agronomic active season. We thereby compared three agricultural landscapes with different crops and different chemical crop protection intensity over a period of five years. We were able to show the changes during the seasons, and the range of pesticide concentrations within the months, as well as the changing pesticide combinations.

Remarkable differences in residue levels between sites depending on the agricultural intensity were found. Surprisingly at the “meadow” site with huge portions of grassland and meadow orchards we found different pesticides in all observation years. However, in comparison to the other sites it showed the lowest intensity of pesticide use. We found many samples without any detectable contamination and the few positive samples only with low concentrations of pesticides. The “grain” site shows already a higher pesticide use with greater portions of grains and maize in the surrounding fields. More substances and higher concentrations of pesticides were measured, especially in spring months. Finally, the “fruit” site, with high portions of permanent crops, such as grapes, is the site with the highest pesticide loads, throughout all months and years of monitoring and 100% contaminated samples, although this site was sampled only three years.

Even though we found the highest concentrations at “fruit”, in comparison with other studies, these concentrations are still on a low level. The highest concentrations found in our study, also being in mg/kg, were up to 14 times lower compared to the maximum concentrations reported by Stoner and Eitzer [31], Traynor et al. [24] and Mullin et al. [23], where pesticide concentrations exceeded 10,000, 20,000 and even 90,000 μg/kg. This is in accordance with an evaluation by Johnson et al. [22] who affirms that it is not unusual to find mg/kg residue levels in hive matrices or collected goods of honeybees when foraging in conventionally farmed land or as pollinators in monocultures with no alternative flowers.

Consequently, PHQ calculations reflected the same image. The higher the cultivation intensity of a field, the more pesticides are used, considering frequency of applications and amount of different substances used. Hence, PHQ are higher at sites with more chemical crop treatments, due to higher concentrations and a higher frequency of comparatively more bee toxic substances. Accordingly, the concentrations measured at the “meadow” site and the toxicity of the detected substances resulted in lower PHQ values, not even coming up the relevant threshold of PHQ = 50. PHQ of 50 was exceeded only at “grain” and fruit”, when substances are categorized harmful to honeybees (methiocarb, dimethoate, clothianidin, imidacloprid) or when measured concentrations were in mg/kg (fluazifop-butyl, dimethomorph, fenhexamid). In general, PHQ values calculated from our pollen samples, are low compared to other studies. Here, PHQs of 500 are exceeded only four times, with 600 being the highest. Traynor et al. [24] calculated in bee bread samples from citrus a PHQ of > 2,000, McArt et al. [57] calculated from beebread from apple orchards a PHQ > 4,000, and Stoner and Eitzer [31] calculated maximum PHQ values of > 40,000.

However, to estimate the possible toxicity for bees, PHQs are only estimates. Stoner and Eitzer [31] were concerned that the picture might be misleading, as honeybee larvae are exposed to more pollen in a very susceptible stage of development [35]. For example, the insecticide fenoxycarb has a low toxicity for adult bees (LD_50_ = 200 μg/bee) which sums up for a low PHQ. Yet studies have shown, that this pesticide is very toxic when ingested by larvae [58–61]. Also, Traynor et al. [24] noted, that PHQ calculations are only simplistic reflections, as pesticide interactions, such as synergism or antagonism are not taken into account, as well as potential metabolism or detoxification of the substances. For this reason, Sanchez-Bayo and Goka [62] included also synergistic effects and frequency of pesticide occurrence into their risk calculations.

Synergism or antagonism of substances may occur, when different substances are exposed to honeybees at the same time. Here, too, we found multiple different substances in one sample, adding up to dozens of different substances during the year inside the hive, as other authors reported previously [23,24,33,34]. Negative side-effects have been proven for e.g. tank mixtures of pyrethroids with sterol biosynthesis inhibiting fungicides (SBI—fungicides) or SBI—fungicides and neonicotinoids[63–68].

In addition to synergistic or additive effects, side-effects by sublethal pesticide concentrations are possible as well. Even though we have shown, that pesticide concentrations exceeded mg/kg in few samples, pesticide detections occurred in sublethal concentrations. Sublethal effects on pesticides, i.e. insecticides have been investigated in honeybees or other pollinators. It is known, that behavior and cognitive performance is impaired, such as learning, memory, homing or flight behavior [36,37,69–73]. In addition, there are changes in biochemistry, growth and development, gene expression [39–42,74,75] or social interactions [38,76]. Even though most effects are determined in worker bees, effects on queen fecundity or sperm viability are reported, too [77,78].

However, as eusocial honeybee colonies are considered to be a superorgansm, they are able to tolerate or buffer stressors such as pesticides or sublethal effects better in comparison to solitary living pollinators [79]. Wild bees use few pollen pellets for provisioning single larvae and these consume pollen directly without a nurse bee in between. Nonetheless, residue analysis of single color fractions revealed sometimes even higher concentrations in certain plant species in comparison to the whole daily collected pollen sample. Generally speaking, pollen of single crops, such as grapes (*V*. *vinifera*) or *Brassica* sp. show more pesticides and higher residue values. Furthermore, pesticides found in pollen pellets of trees such as horse chestnut (*Aesculus hippocastanum* L.), maple (*Acer*) or false acacia (*R*. *pseudoacacia*) or wild flowers and companion plants, respectively, indicate applications during flowering or drift. In addition, from high pesticide concentrations in weed species, such as *Achillea*, direct applications in the field, aiming probably towards fungal diseases in grains and *Achillea* may be inferred. Hence, solitary bees, that only use small amounts of a certain pollen source for the provision of their nests, are generally more endangered in such intensively cultivated agroecosystems [9,80]. Wild bees not only contribute to biodiversity but are also important indicators of environmental health and should not be harmed by pesticides [5,81]. Therefore, not only honeybees but other species of bees should be taken into consideration when crop protecting substances are tested for registration and risk assessments of pesticides should be revised [82].

Not only in the daily collected samples but also in their color sorted fractions, we found 12 substances that are either categorized harmful to honeybees or are not supposed to be exposed to honeybees. For example, picaridin is not a crop protection agent, rather than a common ingredient of commercially available insect repellents. On inquiry, the beekeeper confirmed using a repellent to keep away ticks when working outside at the apiary. This substance on legs and arms probably could contaminate the pollen load during pollen collection. However, this compound is not known to be harmful to honeybees [83]. Another example is seed coating, where an exposure to bees is not expected. However, during sowing process, substances used for seed treatments may be abraded, emitted by machinery and cause dust-drift to adjacent flowering plants [12,84]. This could explain the frequent detection of methiocarb, fuberidazole or pencycuron in our spring samples at the time of sowing. Two other substances, coumaphos and thymol, are registered as varroacides for in-hive applications. These substances might volatilize inside the hive and settle on the hairs of the honeybee. When combing through the hair while making pollen packages the substances could be incorporated into the pollen loads. Toxic neonictinoids, i.e. clothianidin and imidacloprid, have been found at the “fruit” site as well a few times with a maximum concentration of 2.4 μg/kg and therefore also below the level considered for acute honey bee toxicity. Our data do not reveal a clear source for the contamination of pollen with these systemic pesticides. As these neonicotinoids are persistent in soil, water and non-target plants, the accumulation in the environment from earlier use cannot be excluded [19,85,86]. Pollen from oilseed rape may be contaminated. However, clothianidin was not found in *Brassica* sp. sub-fractions in our analyses. Dust abrading from seed treated fodder beet or beet root, which is sown at that time, seems unlikely due to abrasion-proof seed pilleting, but can also not be excluded. We can only speculate, from which plants these detections originate, and further in-depth analyses should be conducted. As clothianidin is registered as spray application in some crops, an unintentional contamination of flowering weeds or shrubs is imaginable. Thus, single contaminated pollen loads might have contaminated the composite sample.

“Cocktails” of pesticides in pollen might raise concern for human health, too, as pollen pellets are used as nutritional supplements [87]. Looking at maximum residue limits for apicultural products, single samples at every site exceeded the limits for some substances leading to an exclusion of the respective pollen samples from further sales. MRL are based on residue data when pesticides are applied correctly according to use instructions [54]. Nonetheless, directly collected pollen by honeybees in the field from treated plants were not considered when thresholds for apicultural products were defined. Therefore, the current agricultural practice makes it impossible for beekeepers to merchandise pollen collected in intensive landscapes–independent of whether there is an impact on human health or not.

Nonetheless, concluding from the majority of concentrations and pesticides found, we assume no misuse of pesticides by the farmers at our three sites and in the observation period, which would lead to direct lethal effects. However, further improvements of seed coatings or sowing technique should be considered. In any case, flowering shrubs, weeds or ruderal plants should be considered while applying chemicals, as well as drift of substances to flowering plants nearby.

The study at hand supports results of other researchers handling pesticide residues in pollen or bee bread samples. We further contribute more details on changes on daily and monthly levels of pesticide exposure. As the detected pesticide concentrations are in sub-lethal levels, we find no reason to believe, that the data published here are to be blamed for higher winter colony losses. Nonetheless more research is necessary in this regard, as they raise concern on chronic effects of these sub-lethal concentrations as well as effects of pesticide mixtures or interactions of pesticides with diseases or pathogens. Some studies already dealt with these questions [45,88–91] even though most investigations still engage in only one endpoint and not in a combination of all three aspects. However, further research needs to be done in order to assess possible risks of chronic ingestion of pesticide mixtures in sub-lethal concentrations on honeybees and other pollinators. Our study gives detailed information to be used to design experiments based on field realistic data.

## Supporting information

S1 Tables **A-C. Cultivated crops at the apiaries.** Acreage of cultivated crops (ha) in a certain area around the apiaries.
(DOCX)

S2 Tables **A-C. Meteorological data.** Mean temperature (°C ± deviation from long-term average (K)) and sum of precipitation (mm ± deviation from long-term average (%)) for each collection month.
(DOCX)

S3 Tables **A-C. Site specific summary.** Overview of substances detected in pollen load samples at the three different sites during the 2012–2016 observation period, assorted according their respective maximum PHQ value.
(DOCX)

S4 Tables Detailed pesticide residue data for each site and year. Pesticide concentrations are given according to LOD/LOQ as obtained by the respective laboratory.
(XLSX)

S5 Tables **A-I. In depth residue analysis of single days.** Pollen pellets of composite samples were sorted according to pellet color into sub-fractions. Pesticide concentrations are given for the composite sample, as well as for each fraction (μg/kg). For each fraction, the dominating plant species is given.
(DOCX)

## References

[1] Cooper J, Dobson H. The benefits of pesticides to mankind and the environment. Crop Prot. 2007;26: 1337–1348. doi: 10.1016/j.cropro.2007.03.022

[2] Oerke EC. Crop losses to pests. J Agric Sci. 2006;144: 31–43. doi: 10.1017/S0021859605005708

[3] Godfray HCJ. The challenge of feeding 9–10 billion people equitably and sustainably. J Agric Sci. 2014;152: 2–8. doi: 10.1017/S0021859613000774

[4] Klein A-M, Vaissière BE, Cane JH, Steffan-Dewenter I, Cunningham SA, Kremen C, et al Importance of pollinators in changing landscapes for world crops. Proc Biol Sci. 2007;274: 303–313. doi: 10.1098/rspb.2006.3721 17164193 10.1098/rspb.2006.3721 PMC1702377

[5] Bartomeus I, Potts SG, Steffan-Dewenter I, Vaissière BE, Woyciechowski M, Krewenka KM, et al Contribution of insect pollinators to crop yield and quality varies with agricultural intensification. PeerJ. 2014;2: 1–20. doi: 10.7717/peerj.328 24749007 10.7717/peerj.328 PMC3976118

[6] Gallai N, Salles JM, Settele J, Vaissière BE. Economic valuation of the vulnerability of world agriculture confronted with pollinator decline. Ecol Econ. 2009;68: 810–821. doi: 10.1016/j.ecolecon.2008.06.014

[7] Lautenbach S, Seppelt R, Liebscher J, Dormann CF. Spatial and temporal trends of global pollination benefit. PLOS one. 2012;7(4): e35954 doi: 10.1371/journal.pone.0035954 22563427 10.1371/journal.pone.0035954 PMC3338563

[8] Tscharntke T, Klein AM, Kruess A, Steffan-Dewenter I, Thies C. Landscape perspectives on agricultural intensification and biodiversity—ecosystem service management. Ecol Lett. 2005;8: 857–874.

[9] Botías C, David A, Hill EM, Goulson D. Contamination of wild plants near neonicotinoid seed-treated crops, and implications for non-target insects. Sci Total Environ. 2016;566–567: 269–278. doi: 10.1016/j.scitotenv.2016.05.065 27220104 10.1016/j.scitotenv.2016.05.065

[10] Aktar W, Sengupta D, Chowdhury A. Impact of pesticides use in agriculture: their benefits and hazards. Interdiscip Toxicol. 2009;2: 1–12. doi: 10.2478/v10102-009-0001-7 21217838 10.2478/v10102-009-0001-7 PMC2984095

[11] Koch H, Weißer P. Exposure of honey bees during pesticide application under field conditions. Apidologie. 1997;28: 439–447.

[12] Schnier HF, Wenig G, Laubert F, Simon V, Schmuck R. Honey bee safety of imidacloprid corn seed treatment. Bull Insectology. 2003;56: 73–75.

[13] Tapparo A, Marton D, Giorio C, Zanella A, Soldà L, Marzaro M, et al Assessment of the environmental exposure of honeybees to particulate matter containing neonicotinoid insecticides coming from corn coated seeds. Environ Sci Technol. 2012;46: 2592–2599. doi: 10.1021/es2035152 22292570 10.1021/es2035152

[14] Samson-Robert O, Labrie G, Chagnon M, Fournier V. Neonicotinoid-contaminated puddles of water represent a risk of intoxication for honey bees. PLoS One. 2014;9: 1–17. doi: 10.1371/journal.pone.0108443 25438051 10.1371/journal.pone.0108443 PMC4249843

[15] Geoghegan T, Kimberly J, Scheringer M. Predicting honeybee exposure to pesticides from vapour drift using a combined pesticide emission and atmospheric transport model. SETAC Australasia—Multidisciplinary approaches to managing environmental pollution. Melbourne: 2013, p. 174.

[16] Rolke D, Persigehl M, Peters B, Sterk G, Blenau W. Large-scale monitoring of effects of clothianidin-dressed oilseed rape seeds on pollinating insects in northern Germany: residues of clothianidin in pollen, nectar and honey. Ecotoxicology. 2016;25: 1691–1701. doi: 10.1007/s10646-016-1723-x 27650369 10.1007/s10646-016-1723-x PMC5093202

[17] Girolami V, Mazzon L, Squartini A, Mori N, Marzaro M, Di Bernardo A, et al Translocation of neonicotinoid insecticides from coated seeds to seedling guttation drops: a novel way of intoxication for bees. J Econ Entomol. 2009;102: 1808–1815. doi: 10.1603/029.102.0511 19886445 10.1603/029.102.0511

[18] Reetz JE, Zühlke S, Spiteller M, Wallner K. Neonicotinoid insecticides translocated in guttated droplets of seed-treated maize and wheat: A threat to honeybees? Apidologie. 2011;42: 596–606. doi: 10.1007/s13592-011-0049-1

[19] Botías C, David A, Horwood J, Abdul-Sada A, Nicholls E, Hill E, et al Neonicotinoid residues in wildflowers, a potential route of chronic exposure for bees. Environ Sci Technol. 2015;49: 12731–12740. doi: 10.1021/acs.est.5b03459 26439915 10.1021/acs.est.5b03459

[20] Bogdanov S. Contaminants of bee products. Apidologie. 2006;37: 1–18. doi: 10.1051/apido

[21] Böhme F, Bischoff G, Zebitz CP, Rosenkranz P, Wallner K. From field to food—Will pesticide contaminated pollen diet lead to a contamination of royal jelly? Apidologie. 2018;49(1): 112–119. doi: 10.1007/s13592-017-0533-3

[22] Johnson RM, Ellis MD, Mullin CA, Frazier M. Pesticides and honey bee toxicity–USA. Apidologie. 2010;41: 312–331. doi: 10.1051/apido/2010018

[23] Mullin CA, Frazier M, Frazier JL, Ashcraft S, Simonds R, vanEngelsdorp D, et al High levels of miticides and agrochemicals in North American apiaries: implications for honey bee health. PLoS One. 2010;5(3): e9754 doi: 10.1371/journal.pone.0009754 20333298 10.1371/journal.pone.0009754 PMC2841636

[24] Traynor KS, Pettis JS, Tarpy DR, Mullin CA, Frazier JL, Frazier M, et al Inhive pesticide exposome: Assessing risks to migratory honey bees from inhive pesticide contamination in the Eastern United States. Nat Sci Reports. 2016;6: 1–16. doi: 10.1038/srep33207 27628343 10.1038/srep33207 PMC5024099

[25] Genersch E, von der Ohe W, Kaatz H, Schroeder A, Otten C, Büchler R, et al The German bee monitoring project: A long term study to understand periodically high winter losses of honey bee colonies. Apidologie. 2010;41: 332–352. doi: 10.1051/apido/2010014

[26] Smodiš Škerl MI, Velikonja Bolta S, Basa Cesnik H, Gregorc A. Residues of Pesticides in honeybee (*Apis mellifera carnica*) bee bread and in pollen loads from treated apple orchards. Bull Environ Contam Toxicol. 2009;83: 374–377. doi: 10.1007/s00128-009-9762-0 19434347 10.1007/s00128-009-9762-0

[27] Orantes-Bermejo FJ, Pajuelo AG, Megías MM, Fernández-Píñar CT. Pesticide residues in beeswax and beebread samples collected from honey bee colonies (*Apis mellifera* L.) in Spain. Possible implications for bee losses. J Apic Res. 2010;48: 243–250. doi: 10.3896/IBRA.1.49.3.03

[28] Porrini C, Mutinelli F, Bortolotti L, Granato A, Laurenson L, Roberts K, et al The status of honey bee health in Italy: Results from the nationwide bee monitoring network. PLoS One. 2016;11: 1–22. doi: 10.1371/journal.pone.0155411 27182604 10.1371/journal.pone.0155411 PMC4868308

[29] Chauzat M-P, Carpentier P, Martel A-C, Bougeard S, Cougoule N, Porta P, et al Influence of pesticide residues on honey bee (Hymenoptera: Apidae) colony health in France. Environ Entomol. 2009;38: 514–23. doi: 10.1603/022.038.0302 19508759 10.1603/022.038.0302

[30] Chauzat M-P, Faucon J-P, Martel A-C, Lachaize J, Cougoule N, Aubert M. A survey of pesticide residues in pollen loads collected by honey bees in France. J Econ Entomol. 2006;99: 253–62. doi: 10.1603/0022-0493-99.2.253 16686121 10.1603/0022-0493-99.2.253

[31] Stoner KA, Eitzer BD. Using a hazard quotient to evaluate pesticide residues detected in pollen trapped from honey bees (*Apis mellifera*) in Connecticut. PLoS One. 2013;8: 1–10. doi: 10.1371/journal.pone.0077550 24143241 10.1371/journal.pone.0077550 PMC3797043

[32] Lambert O, Piroux M, Puyo S, Thorin C, L’Hostis M, Wiest L, et al Widespread occurrence of chemical residues in beehive matrices from apiaries located in different landscapes of Western France. PLoS One. 2013;8: 1–12. doi: 10.1371/journal.pone.0067007 23799139 10.1371/journal.pone.0067007 PMC3684584

[33] Pettis JS, Lichtenberg EM, Andree M, Stitzinger J, Rose R. Crop pollination exposes honey bees to pesticides which alters their susceptibility to the gut pathogen *Nosema ceranae*. PLoS One. 2013;8: 1–9. doi: 10.1371/journal.pone.0070182 23894612 10.1371/journal.pone.0070182 PMC3722151

[34] Tosi S, Costa C, Vesco U, Quaglia G, Guido G. A 3-year survey of Italian honey bee-collected pollen reveals widespread contamination by agricultural pesticides. Sci Total Environ. 2018;615: 208–218. doi: 10.1016/j.scitotenv.2017.09.226 28968582 10.1016/j.scitotenv.2017.09.226

[35] Rortais A, Arnold G, Halm M-P, Touffet-Briens F. Modes of honeybees exposure to systemic insecticides: estimated amounts of contaminated pollen and nectar consumed by different categories of bees. Apidologie. 2005;36: 71–83. doi: 10.1051/apido

[36] Andrione M, Vallortigara G, Antolini R, Haase A. Neonicotinoid-induced impairment of odour coding in the honeybee. Sci Rep. 2016;6: 38110 doi: 10.1038/srep38110 27905515 10.1038/srep38110 PMC5131477

[37] Tosi S, Burgio G, Nieh JC. A common neonicotinoid pesticide, thiamethoxam, impairs honey bee flight ability. Sci Rep. 2017;7: 1201 doi: 10.1038/s41598-017-01361-8 28446783 10.1038/s41598-017-01361-8 PMC5430654

[38] Medrzycki P, Montanari R, Bortolotti L, Sabatini AG, Maini S, Porrini C. Effects of imidacloprid administered in sub-lethal doses on honey bee behaviour. Laboratory tests. Bull Insectology. 2003;56: 59–62.

[39] Alkassab AT, Kirchner WH. Sublethal exposure to neonicotinoids and related side effects on insect pollinators: honeybees, bumblebees, and solitary bees. J Plant Dis Prot. 2016;124(1). doi: 10.1007/s41348-016-0041-0

[40] Dai PL, Wang Q, Sun JH, Liu F, Wang X, Wu YY, et al Effects of sublethal concentrations of bifenthrin and deltamethrin on fecundity, growth, and development of the honeybee *Apis mellifera ligustica*. Environ Toxicol Chem. 2010;29: 644–649. doi: 10.1002/etc.67 20821489 10.1002/etc.67

[41] Brandt A, Gorenflo A, Siede R, Meixner M, Büchler R. The neonicotinoids thiacloprid, imidacloprid, and clothianidin affect the immunocompetence of honey bees (*Apis mellifera* L.). J Insect Physiol. 2016;86: 40–47. doi: 10.1016/j.jinsphys.2016.01.001 26776096 10.1016/j.jinsphys.2016.01.001

[42] Wu MC, Chang YW, Lu KH, Yang EC. Gene expression changes in honey bees induced by sublethal imidacloprid exposure during the larval stage. Insect Biochem Mol Biol. 2017;88: 12–20. doi: 10.1016/j.ibmb.2017.06.016 28732753 10.1016/j.ibmb.2017.06.016

[43] Desneux N, Decourtye A, Delpuech J-M. The sublethal effects of pesticides on beneficial arthropods. Annu Rev Entomol. 2007;52: 81–106. doi: 10.1146/annurev.ento.52.110405.091440 16842032 10.1146/annurev.ento.52.110405.091440

[44] Anastassiades M, Lehotay SJ, Stajnbaher D, Schenck FJ. Fast and easy multiresidue method employing acetonitrile extraction/partitioning and “dispersive solid-phase extraction” for the determination of pesticide residues in produce. J AOAC Int. 2003;86: 412–431. 12723926

[45] Böhme F, Bischoff G, Zebitz CP, Rosenkranz P, Wallner K. Chronic exposure of honeybees, *Apis mellifera* (Hymenoptera: Apidae), to a pesticide mixture in realistic field exposure rates. Apidologie. 2017;48: 353–363. doi: 10.1007/s13592-016-0479-x

[46] PPDB—Pesticide Properties DataBase, Univesity of Hertfordshire, 2017. Available from: http://sitem.herts.ac.uk/aeru/ppdb/en/atoz.htm#A. Accessed on 2017-02-20.

[47] US EPA. United States Environmental Protection Agency ecotoxicology database 2017. Available from: https://cfpub.epa.gov/ecotox/. Accessed on 2017-04-10.

[48] Agence nationale de sécurité sanitaire agritox database 2017. Available from: http://www.agritox.anses.fr/guides/guide-agritox-anglais.html. Accessed on 2017-04-10.

[49] Dahlgren L, Johnson RM, Siegfried BD, Ellis MD. Comparative toxicity of acaricides to honey bee (Hymenoptera: Apidae) workers and queens. J Econ Entomol. 2012;105: 1895–1902. 23356051 10.1603/ec12175

[50] FRAC Code List. Fungicide Resistance Action Committee 2017. Available from: http://www.frac.info/publications/downloads. Accessed on 2017-05-06.

[51] IRAC Classification Scheme. Insecticide Resistance Action Committee 2017. Available from: http://www.irac-online.org/modes-of-action/. Accessed on 2017-05-06.

[52] HRAC. Herbicide Resistance Action Committee 2017. Available from: http://www.hracglobal.com/. Accessed on 2017-05-06.

[53] Federal Office of Consumer Protection and Food Safety. Authorization procedure for plant protection products. 2017. Available from: http://www.bvl.bund.de/EN/04_PlantProtectionProducts/03_Applicants/04_AuthorisationProcedure/08_Environment/ppp_bee_protection_node.html#doc8564726bodyText3. Accessed on 2017-05-19.

[54] European Commission. EU Pesticides Database. 2017. Available from: http://ec.europa.eu/food/plant/pesticides/eu-pesticides-database/public/?event=homepage&language=EN. Accessed on 2017-04-26.

[55] US EPA. New Pesticide Fact Sheet—Picaridin, United States Environmental Protection Agency, Office of Prevention, Pesticides and Toxic Substances, Office of Pesticide Programs, U.S. Government Printing Office: Washington, DC. 2005.

[56] NPIC. Nacional Pesticide Information Center, Picaridin Technical Fact Sheet, Oregon State University 2017. Available from: http://npic.orst.edu/factsheets/archive/Picaridintech.html#references. Accessed on 2017-05-09.

[57] McArt SH, Fersch AA, Milano NJ, Truitt LL, Böröczky K. High pesticide risk to honey bees despite low focal crop pollen collection during pollination of a mass blooming crop. Nat Sci Reports. 2017;7: 1–10. doi: 10.1038/srep46554 28422139 10.1038/srep46554 PMC5396195

[58] de Ruijter A, van der Steen J. Feldversuche zum Effekt einer Insegar (Fenoxycarb)- Spritzung während der Apfelblüte auf die Honigbiene. Apidologie. 1987;18: 355–357.

[59] Czoppelt C. Toxizitätsmessungen mit dem Juvenoid Fenoxycarb an Bienenlarven im in vitro Aufzuchttest. Apidologie. 1991;22: 457–459.

[60] Aupinel P, Fortini D, Michaud B, Marolleau F, Tasei J-N, Odoux J-F. Toxicity of dimethoate and fenoxycarb to honey bee brood (*Apis mellifera*), using a new in vitro standardized feeding method. Pest Manag Sci. 2007;63: 1090–1094. doi: 10.1002/ps.1446 17879979 10.1002/ps.1446

[61] Tasei J. Effects of insect growth regulators on honey bees and non-Apis bees. A review. Apidologie. 2001;32: 527–245. doi: 10.1051/apido:2001102

[62] Sanchez-Bayo F, Goka K. Pesticide residues and bees—A risk assessment. PLoS One. 2014;9: 1–16. doi: 10.1371/journal.pone.0094482 24718419 10.1371/journal.pone.0094482 PMC3981812

[63] Federal Office of Consumer Protection and Food Safety. Online data base on plant protection products. 2017. Available from: https://apps2.bvl.bund.de/psm/jsp/index.jsp. Accessed on 2017-04-26.

[64] Pillings ED, Bromley-Challenor KAC, Walker CH, Jepson PC. Mechanism of synergism between the pyrethroid insecticide and the imidazole fungicide prochloraz in the honeybee (*Apis mellifera* L.). Pestic Biochem. 1995;51: 1–11.

[65] Pillings ED, Jepson DC. Synergism between EBI fungicides and pyrethoid insecticide in the honeybee (*Apis mellifera*). Pestic Sci. 1993;39: 293–297.

[66] Thompson HM, Fryday SL, Harkin S, Milner S. Potential impacts of synergism in honeybees (*Apis mellifera*) of exposure to neonicotinoids and sprayed fungicides in crops. Apidologie. 2014;45: 545–553. doi: 10.1007/s13592-014-0273-6

[67] Iwasa T, Motoyama N, Ambrose JT, Roe RM. Mechanism for the differential toxicity of neonicotinoid insecticides in the honey bee, *Apis mellifera*. Crop Prot. 2004;23: 371–378. doi: 10.1016/j.cropro.2003.08.018

[68] Colin ME, Belzunces LP. Evidence of synergy between prochloraz and deltametrin in *Apis mellifera* L.: A convenient biological approach. Pestic Sci. 1992;36: 115–119.

[69] Teeters BS, Johnson RM, Ellis MD, Siegfried BD. Using video-tracking to assess sublethal effects of pesticides on honey bees (*Apis mellifera* L.). Environ Toxicol Chem. 2012;31: 1349–1354. doi: 10.1002/etc.1830 22488825 10.1002/etc.1830

[70] Decourtye A, Devillers J, Genecque E, Le Menach K, Budzinski H, Cluzeau S, et al Comparative sublethal toxicity of nine pesticides on olfactory learning performances of the honeybee *Apis mellifera*. Arch Environ Contam Toxicol. 2005;48: 242–250. doi: 10.1007/s00244-003-0262-7 15750780 10.1007/s00244-003-0262-7

[71] Fischer J, Müller T, Spatz AK, Greggers U, Grünewald B, Menzel R. Neonicotinoids interfere with specific components of navigation in honeybees. PLoS One. 2014;9: 1–10. doi: 10.1371/journal.pone.0091364 24646521 10.1371/journal.pone.0091364 PMC3960126

[72] Urlacher E, Monchanin C, Rivière C, Richard FJ, Lombardi C, Michelsen-Heath S, et al Measurements of chlorpyrifos levels in forager bees and comparison with levels that disrupt honey bee odor-mediated learning under laboratory conditions. J Chem Ecol. 2016;42: 127–138. doi: 10.1007/s10886-016-0672-4 26872472 10.1007/s10886-016-0672-4

[73] Smagghe G, Deknopper J, Meeus I, Mammaerts V. Dietary chlorantraniliprole suppresses reproduction in worker bumblebees. Pest Manag Sci. 2013;69: 787–791. doi: 10.1002/ps.3504 23564706 10.1002/ps.3504

[74] Wu JY, Anelli CM, Sheppard WS. Sub-lethal effects of pesticide residues in brood comb on worker honey bee (*Apis mellifera*) development and longevity. PLoS One. 2011;6(2): 1–11. doi: 10.1371/journal.pone.0014720 21373182 10.1371/journal.pone.0014720 PMC3044129

[75] Williamson SM, Moffat C, Gomersall MAE, Saranzewa N, Connolly CN, Wright GA. Exposure to acetylcholinesterase inhibitors alters the physiology and motor function of honeybees. Front Physiol. 2013;4: 1–10. doi: 10.3389/fphys.2013.00001 23386834 10.3389/fphys.2013.00013 PMC3564010

[76] Forfert N, Moritz RFA. Thiacloprid alters social interactions among honey bee workers (*Apis mellifera*). J Apic Res. 2017;56: 467–474. doi: 10.1080/00218839.2017.1332542

[77] Wu-Smart J, Spivak M. Sub-lethal effects of dietary neonicotinoid insecticide exposure on honey bee queen fecundity and colony development. Sci Rep. 2016;6: 32108 doi: 10.1038/srep32108 27562025 10.1038/srep32108 PMC4999797

[78] Chaimanee V, Evans J, Chen Y, Jackson C, Pettis J. Sperm viability and gene expression in honey bee queens (*Apis mellifera*) following exposure to the neonicotinoid insecticide imidacloprid and the organophosphate acaricide coumaphos. J Insect Physiol. 2016;89: 1–8. doi: 10.1016/j.jinsphys.2016.03.004 26979384 10.1016/j.jinsphys.2016.03.004

[79] Straub L, Williams GR, Pettis J, Fries I, Neumann P. Superorganism resilience: Eusociality and susceptibility of ecosystem service providing insects to stressors. Curr Opin Insect Sci. 2015;12: 109–112. doi: 10.1016/j.cois.2015.10.010

[80] Rundlöf M, Andersson GKS, Bommarco R, Fries I, Hederström V, Herbertsson L, et al Seed coating with a neonicotinoid insecticide negatively affects wild bees. Nature. 2015;521(7550): 77–80. doi: 10.1038/nature14420 25901681 10.1038/nature14420

[81] Tscharntke T, Klein AM, Kruess A, Steffan-Dewenter I, Thies C. Landscape perspectives on agricultural intensification and biodiversity—ecosystem service management. Ecol Lett. 2005;8: 857–874.

[82] Decourtye A, Henry M, Desneux N. Overhaul pesticide testing on bees. Nature. 2013;497(7448): 188. 10.1038/497188a 23657341

[83] Rosenkranz P, Von Der Ohe W, Schäfer M, Genersch E, Büchler R, Berg S, et al. Deutsches Bienenmonitoring - „DeBiMo“, Zwischenbericht, Projektzeitraum: 01/2014–12/2014 2014. Available from: https://bienenmonitoring.uni-hohenheim.de/fileadmin/einrichtungen/bienenmonitoring/Dokumente/Zwischenbericht_DeBiMo_1-12_2014.pdf Accessed on 2017-05-24.

[84] Pistorius J, Bischoff G, Heimbach U, Stähler M. Bee poisoning incidents in Germany in spring 2008 caused by abrasion of active substance from treated seeds during sowing of maize. Julius-Kühn-Archiv 2010;423: 118–126.

[85] Schaafsma A, Limay-Rios V, Xue Y, Smith J, Baute T. Field-scale examination of neonicotinoid insecticide persistence in soil as a result of seed treatment use in commercial maize (corn) fields in southwestern Ontario. Environ Toxicol Chem. 2016;35: 295–302. doi: 10.1002/etc.3231 26332416 10.1002/etc.3231

[86] Bonmatin JM, Giorio C, Girolami V, Goulson D, Kreutzweiser DP, Krupke C, et al Environmental fate and exposure; neonicotinoids and fipronil. Environ Sci Pollut Res. 2015;22: 35–67. doi: 10.1007/s11356-014-3332-7 25096486 10.1007/s11356-014-3332-7 PMC4284396

[87] Ghosh S, Jung C. Nutritional value of bee-collected pollens of hardy kiwi, *Actinidia arguta* (Actinidiaceae) and oak, *Quercus* sp. (Fagaceae). J Asia Pac Entomol. 2017;20: 245–251.

[88] Gill RJ, Raine NE. Chronic impairment of bumblebee natural foraging behaviour induced by sublethal pesticide exposure. Funct Ecol. 2014;28: 1459–1471. doi: 10.1111/1365-2435.12292

[89] Gill RJ, Ramos-Rodriguez O, Raine NE. Combined pesticide exposure severely affects individual- and colony-level traits in bees. Nature. 2012;490: 105–108. doi: 10.1038/nature11585 10.1038/nature11585 PMC3495159 23086150

[90] Zhu W, Schmehl DR, Mullin CA, Frazier JL. Four common pesticides, their mixtures and a formulation solvent in the hive environment have high oral toxicity to honey bee larvae. PLoS One. 2014;9(1): e77547 doi: 10.1371/journal.pone.0077547 24416121 10.1371/journal.pone.0077547 PMC3885384

[91] Sánchez-Bayo F, Goulson D, Pennacchio F, Nazzi F, Goka K, Desneux N. Are bee diseases linked to pesticides?–A brief review. Environ Int. 2016;89–90: 7–11. doi: 10.1016/j.envint.2016.01.009 26826357 10.1016/j.envint.2016.01.009

