# Supplementary material for: Pesticide residue survey of pollen loads collected by honeybees (Apis mellifera) in daily intervals at three agricultural sites in South Germany
Source: PLoS One. 2018 Jul 6;13(7):e0199995. doi: 10.1371/journal.pone.0199995 (PMC6034819; doi:10.1371/journal.pone.0199995)
Supplement: S1 Tables — A-C. Cultivated crops at the apiaries. Acreage of cultivated crops (ha) in a certain area around the apiaries. (DOCX) [file pone.0199995.s001.docx]

**S1 Table A. Cultivated crops at the apiary.** Acreage of cultivated crops (ha) in an area of 40 km^2^ (radius of 3.6 km) around the apiary “meadow”. Data for 2016 not yet available.

| **Crop** | **2012** | **2013** | **2014** | **2015** |
| --- | --- | --- | --- | --- |
| **Cereals** | 644.9 | 643.1 | 629.3 | 618.1 |
| **Protein crops** | 2.4 | 2.3 | 2.8 | 21.1 |
| **Oilseed crops** | 37.8 | 40.2 | 28.2 | 40.3 |
| **Forage crops** | 437.0 | 432.0 | 462.8 | 435.9 |
| **Permanent grassland** | 2389.4 | 2338.4 | 2352.6 | 2380.8 |
| **Root crops** | 2.2 | 1.8 | 1.4 | 1.1 |
| **Ornamental flowers** | 0.6 | 0.3 | - | - |
| **Permanent crops** | 3.6 | 5.9 | 4.9 | 4.8 |
| **Strawberries** | - | 32.7 | 28.3 | 33.2 |
| **Other crops** | 13.4 | 13.8 | 14.3 | 17.0 |
| **Forrest** | 484.0 | 489.0 | 382.8 | 369.6 |
| **Yard, paths and buildings** | 55.8 | 53.3 | 55.2 | 54.0 |
| **Miscellaneous areas** | 17.8 | 13.3 | 14.0 | 15.0 |
| **Total ha** | 4088.9 | 4065.3 | 3976.6 | 3990.9 |

**S1 Table B. Cultivated crops at the apiary**. Acreage of cultivated crops (ha) in an area of 51 km^2^ (radius of 4 km) around the apiary “grain”. Data for 2016 not yet available.

| **Crop** | **2012** | **2013** | **2014** | **2015** |
| --- | --- | --- | --- | --- |
| **Cereals** | 2308.5 | 2314.2 | 2300.5 | 2246.5 |
| **Protein crops** | - | 0.5 | 1.6 | 5.4 |
| **Oilseed crops** | 262.4 | 294.0 | 271.9 | 213.7 |
| **Forage crops** | 1175.8 | 1137.7 | 1177.0 | 1255.3 |
| **Permanent grassland** | 1100.1 | 1129.6 | 1142.1 | 1149.5 |
| **Root crops** | 38.72 | 41.1 | 28.3 | 37.7 |
| **Permanent crops** | 34.8 | 32.0 | 34.8 | 34.0 |
| **Other crops** | 2.7 | 0.05 | 0.05 | 0.4 |
| **Forrest** | 116.4 | 116.9 | 117.2 | 117.3 |
| **Yard, paths and buildings** | 54.9 | 55.0 | 55.8 | 55.9 |
| **Miscellaneous areas** | 46.1 | 44.4 | 48.6 | 57.8 |
| **Total ha** | 5140.4 | 5165.5 | 5177.9 | 5173.5 |

**S1 Table C. Cultivated crops at the apiary**. Acreage of cultivated crops (ha) in an area of 34 km^2^ (radius of 3,3 km) around the apiary “fruit”.

| **Crop** | **2012** | **2013** | **2014** |
| --- | --- | --- | --- |
| **Cereals** | 1404.8 | 1464.3 | 1390.4 |
| **Protein crops** | 1.3 | 1.2 | 0.9 |
| **Oilseed crops** | 144.5 | 110.3 | 106.7 |
| **Forage crops** | 79.2 | 81.0 | 117.4 |
| **Permanent grassland** | 295.0 | 304.9 | 294.0 |
| **Root crops** | 303.5 | 279.8 | 324.6 |
| **Ornamental flowers** | 10.8 | 8.7 | 10.1 |
| **Permanent crops** | 952.5 | 958.3 | 964.8 |
| **Strawberries** | 22.7 | 21.79 | 23.3 |
| **Other crops** | 161.2 | 140.8 | 142.3 |
| **Forrest** | 11.3 | 11.3 | 11.3 |
| **Yard, paths and buildings** | 45.2 | 44.8 | 43.6 |
| **Miscellaneous areas** | 49.6 | 46.0 | 56.0 |
| **Total ha** | 3481.6 | 3473.2 | 3485.4 |

Cereals: spelt, barley, oat, wheat, rye, triticale, grain maize, millet

Protein crops: pea, field bean

Oilseed crops: rapeseed, sunflower, flax, soy bean

Forage crops: grass, fodder beet, clover, alfalfa, silage maize

Permanent grassland: pasture, meadow, grassland, meadow orchards, traditional extensive orchards

Root crops: potatoes, sugar beet root, topinambour

Permanent crops: nurseries, pomaceous and stone fruits, christmas trees, soft fruit, asparagus, vineyard area, rhubarb, table grapes

Other crops: miscanthus, vegetable, spice or medicinal plants

Miscellaneous areas: field margins, biotops without agricultural use, kitchen gardens, infertile land, uncultivated land

This information was kindly granted by the Ministry of Rural affairs and Consumer Protection Baden-Württemberg.
