# Supplementary material for: Pesticide residue survey of pollen loads collected by honeybees (Apis mellifera) in daily intervals at three agricultural sites in South Germany
Source: PLoS One. 2018 Jul 6;13(7):e0199995. doi: 10.1371/journal.pone.0199995 (PMC6034819; doi:10.1371/journal.pone.0199995)
Supplement: S2 Tables — A-C. Meteorological data. Mean temperature (°C ± deviation from long-term average (K)) and sum of precipitation (mm ± deviation from long-term average (%)) for each collection month. (DOCX) [file pone.0199995.s002.docx]

**Meteorological data site “meadow”**

**S2 Table A. Meteorological data.** Meteorological data from the weather station at Schlat (434 m a.s.l., 13 km away from the pollen collection site). Mean temperature (°C ± deviation from long-term average (K)) and sum of precipitation (mm ± deviation from long-term average (%)) for each collection month. The long-term average (1961-1990) was taken from a close-by location (Merklingen).

| **Year** | **Month** | **Mean Temperature (°C ± deviation from long-term average (K))** | **Sum of precipitation (mm ± deviation from long-term average (%))** |
| --- | --- | --- | --- |
| **2012** | March | 7.7 (+5.8) | 23.4 (-65.9) |
|  | April | 9.0 (+2.9) | 48.6 (-42.3) |
|  | May | 15.1 (+4.5) | 47.0 (-45.5) |
|  | June | 17.6 (+3.8) | 61.8 (-46.5) |
| **2013** | April | 9.2 (+3.1) | 43.6 (-48.2) |
|  | May | 11.4 (+0.8) | 150.4 (+74.5) |
|  | June | 16.6 (+2.8) | 68.2 (-41.0) |
|  | July | 20.8 (+5.0) | 100.4 (+14.5) |
|  | Aug. | 18.5 (+3.5) | 50.2 (-46.3) |
| **2014** | March | 8.2 (+6.3) | 17.4 (-74.7) |
|  | April | 11.5 (+5.4) | 37.4 (-55.6) |
|  | May | 13.1 (+2.5) | 58.8 (-31.8) |
| **2015** | March | 6.3 (+4.4) | 37.4 (-45.6) |
|  | April | 9.5 (+3.4) | 46.0 (-45.4) |
|  | May | 14.2 (+3.6) | 74.0 (-14.2) |
|  | June | 18.1 (+4.3) | 68.1 (-41.1) |
|  | July | 22.4 (+6.6) | 33.7 (-61.6) |
| **2016** | April | 9.3 (+3.2) | 64.0 (-24.0) |
|  | May | 14.4 (+3.8) | 95.1 (+10.3) |
|  | June | 17.9 (+4.1) | 122.9 (+6.3) |
|  | July | 20.1 (+4.3) | 51.6 (-41.2) |

**Meteorological data site “grain”**

**S2 Table B. Meteorological data.** Meteorological data from the weather station at Biberach an der Riß (533 m a.s.l., 30 km away from the pollen collection site). Mean temperature (°C ± deviation from long-term average (K)) and sum of precipitation (mm ± deviation from long-term average (%)) for each collection month. The long-term average (1961-1990) was taken from a close-by location (Schemmerhofen-Ingerking).

| **Year** | **Month** | **Mean Temperature (°C ± deviation from long-term average (K))** | **Sum of precipitation (mm ± deviation from long-term average (%))** |
| --- | --- | --- | --- |
| **2012** | Feb. | -5.0 (-5.0) | 4.5 (-88.8) |
|  | March | 6.7 (+3.2) | 21.8 (-38.1) |
|  | April | 7.9 (+0.5) | 66.3 (+17.8) |
|  | May | 13.7 (+1.7) | 45.4 (-48.6) |
|  | June | 16.6 (+1.1) | 138.6 (+29.2) |
|  | July | 17.4 (+0.1) | 91.7 (+9.2) |
| **2013** | March | 1.3 (-2.2) | 37.1 (+5.4) |
|  | April | 7.9 (+0.5) | 76.5 (+35.9) |
|  | May | 10.5 (-1.5) | 116.6 (+32.0) |
|  | June | 15.4 (-0.1) | 107.1 (-0.2) |
|  | July | 19.8 (+2.5) | 44.1 (-47.5) |
| **2014** | March | 6.4 (+2.9) | 22.0 (-37.5) |
|  | April | 10.0 (+2.6) | 42.3 (-24.9) |
|  | May | 12.0 (+0.0) | 55.7 (-36.9) |
|  | June | 16.7 (+1.2) | 72.1 (-32.8) |
|  | July | 17.6 (+0.3) | 131.0 (+56.0) |
| **2015** | March | 4.8 (+1.3) | 95.3 (+170.7) |
|  | April | 8.6 (+1.2) | 80.5 (+43.0) |
|  | May | 13.1 (+1.1) | 153.3 (+73.6) |
|  | June | 16.8 (+1.3) | 89.6 (-16.5) |
|  | July | 20.6 (+3.3) | 44.0 (-47.6) |
| **2016** | March | 3.3 (-0.2) | 41.5 (+17.9) |
|  | April | 7.6 (+0.2) | 61.1 (+8.5) |
|  | May | 11.9 (-0.1) | 165.6 (+87.5) |
|  | June | 16.0 (+0.5) | 184.1 (+71.6) |
|  | July | 18.6 (+1.3) | 69.1 (-17.7) |

**Meteorological data site “fruit”**

**S2 Table C. Meteorological data.** Meteorological data from the weather station at Lauffen a.N. (206 m a.s.l., 8 km away from the pollen collection site). Mean temperature (°C ± deviation from long-term average (K)) and sum of precipitation (mm ± deviation from long-term average (%)) for each collection month. The long-term average (1961-1990) was taken from a close-by location (Heilbronn).

| **Year** | **Month** | **Mean Temperature (°C ± deviation from long-term average (K))** | **Sum of precipitation (mm ± deviation from long-term average (%))** |
| --- | --- | --- | --- |
| **2012** | March | 8.7 (+3.0) | 6.0 (-89.0) |
|  | April | 10.0 (+0.4) | 18.5 (+68.2) |
|  | May | 16.6 (+2.6) | 27.0 (-65.9) |
|  | June | 18.2 (+1.1) | 52.3 (-37.2) |
| **2013** | April | 10.3 (+0.7) | 41.5 (-28.7) |
|  | May | 12.6 (-1.4) | 79.9 (+0.9) |
|  | June | 17.7 (+0.6) | 51.7 (-37.9) |
|  | July | 22.0 (+3.1) | 42.0 (-37.8) |
| **2014** | March | 8.8 (+3.1) | 0.0 (-100.0) |
|  | April | 12.8 (+3.2) | 29.4 (-49.5) |
|  | May | 13.8 (-0.2) | 51.0 (-35.6) |
|  | June | 18.7 (+1.6) | 8.7 (-89.6) |

Meteorological data for all three sites were assessed at www.wetter-bw.de, on March 16, ‎2017.
