# Supplementary material for: Pesticide residue survey of pollen loads collected by honeybees (Apis mellifera) in daily intervals at three agricultural sites in South Germany
Source: PLoS One. 2018 Jul 6;13(7):e0199995. doi: 10.1371/journal.pone.0199995 (PMC6034819; doi:10.1371/journal.pone.0199995)
Supplement: S3 Tables — A-C. Site specific summary. Overview of substances detected in pollen load samples at the three different sites during the 2012–2016 observation period, assorted according their respective maximum PHQ value. (DOCX) [file pone.0199995.s003.docx]

**S3 Table A. Site specific summary.** Overview of 24 different substances detected in 82 pollen load samples at the “meadow” site during the 2012-2016 observation period, assorted according their respective maximum PHQ value.

| Substance | Pesticide class ^a)^ | Number of positive detections in 5 years | Number of years detected (out of 5 years) | Lowest detected conc. (µg/kg) ^b)^ | Highest detected conc. (µg/kg) | Mean (± SEM) | 1. quartile | Median | 2. quartile | PHQ_max_ |
| --- | --- | --- | --- | --- | --- | --- | --- | --- | --- | --- |
| Etofenprox | I | 3 | 1 | 3.6 | 6.9 | 5.3 (± 0.8) | - | 5.5 | - | 25.56 |
| Thiacloprid | I | 26 | 4 | 2.6 | 294.6 | 32.5 (± 11.8) | 4.1 | 8.9 | 23.7 | 17.01 |
| Methiocarb | I | 6 | 2 | 2.1 | 4.1 | 2.7 (± 0.3) | 2.2 | 2.5 | 2.9 | 8.82 |
| Dimoxystrobin | F | 9 | 4 | 2.3 | 161.6 | 42.6 (± 17.6) | 3.8 | 7.4 | 74.4 | 2.04 |
| Boscalid | F | 13 | 4 | 2.3 | 75.9 | 23.7 (± 6.7) | 4.2 | 10.8 | 33.1 | 0.76 |
| Fluazifop-butyl | H | 13 | 2 | 2.0 | 41.2 | 16.2 (± 3.4) | 8.6 | 9.9 | 18.3 | 0.65 |
| Prothioconazole-desthio | F | 13 | 3 | 2.9 | 34.5 | 8.4 (± 2.2) | 3.2 | 6.4 | 8.7 | 0.49 |
| Tebuconazole | F | 6 | 2 | 2.5 | 15.8 | 6.8 (± 1.8) | 3.7 | 5.4 | 7.6 | 0.19 |
| Fluopyram | F | 2 | 1 | 6.2 | 19.2 | - | - | - | - | 0.19 |
| Terbuthylazine | H | 1 | 1 | 3.5 | 3.5 | - | - | - | - | 0.16 |
| Azoxystrobin | F | 3 | 2 | 2.3 | 3.8 | 3.1 (± 0.4) | - | 3.1 | - | 0.15 |
| Myclobutanil | F | 1 | 1 | 5.0 | 5.0 | - | - | - | - | 0.15 |
| Acetamiprid | I | 1 | 1 | 2.1 | 2.1 | - | - | - | - | 0.14 |
| Metconazole | F | 8 | 1 | 2.0 | 8.9 | 4.2 (± 0.9) | 2.2 | 3.1 | 5.1 | 0.10 |
| Cyprodinil | F | 9 | 1 | 2.0 | 9.9 | 5.4 (± 1.1) | 2.6 | 4.2 | 9.7 | 0.09 |
| Pyraclostrobin | F | 1 | 1 | 6.4 | 6.4 | - | - | - | - | 0.09 |
| Propiconazole | F | 2 | 2 | 4.1 | 6.5 | - | - | - | - | 0.07 |
| Trifloxystrobin | F | 1 | 1 | 13.2 | 13.2 | - | - | - | - | 0.07 |
| Metolachlor | H | 1 | 1 | 5.4 | 5.4 | - | - | - | - | 0.05 |
| Pencycuron | F | 1 | 1 | 4.5 | 4.5 | - | - | - | - | 0.04 |
| Prosulfocarb | H | 2 | 1 | 2.5 | 3.2 | - | - | - | - | 0.03 |
| Pendimethalin | H | 1 | 1 | 3.2 | 3.2 | - | - | - | - | 0.03 |
| Fluoxastrobin | F | 1 | 1 | 2.0 | 2.0 | - | - | - | - | 0.002 |
| Picaridin | IR | 1 | 1 | 18.7 | 18.7 | - | - | - | - |  |

1. F = fungicide, H = herbicide, I = insecticide, IR = insect repellent
2. Lowest detected concentration according to LOD as obtained by LUFA Speyer, for better comparability of all sites.

**S3 Table B. Site specific summary.** Overview of 37 different substances detected in 143 pollen load samples at the “grain” site during the 2012-2016 observation period, assorted according their respective maximum PHQ value.

| Substance | Pesticide class ^a)^ | Number of positive detections in 5 years | Number of years detected (out of 5 years) | Lowest detected conc. (µg/kg) ^b)^ | Highest detected conc. (µg/kg) | Mean (± SEM) | 1. quartile | Median | 2. quartile | PHQ_max_ |
| --- | --- | --- | --- | --- | --- | --- | --- | --- | --- | --- |
| Dimethoate | I | 1 | 1 | 19.7 | 19.7 | - | - | - | - | 164.41 |
| Methiocarb | I | 21 | 5 | 2.7 | 47.7 | 14.3 (± 2.8) | 3.3 | 8.5 | 20.3 | 101.54 (3x 50+) |
| Thiacloprid | I | 73 | 5 | 2.0 | 364.3 | 56.8 (± 7.1) | 14.0 | 38.5 | 85.0 | 21.04 |
| Boscalid | F | 28 | 5 | 2.2 | 1,496.4 | 65.1 (± 52.1) | 4.2 | 8.2 | 12.8 | 14.96 |
| Fluazifop-butyl | H | 11 | 1 | 13.6 | 871.8 | 289.9 (± 89.0) | 67.0 | 201.8 | 357.2 | 13.84 |
| Azoxystrobin | F | 19 | 5 | 2.2 | 206.8 | 17.7 (± 10.3) | 3.4 | 4.9 | 10.4 | 8.27 |
| Dimoxystrobin | F | 24 | 5 | 2.3 | 576.2 | 35.9 (± 23.4) | 3.5 | 5.3 | 7.9 | 7.26 |
| Methiocarb-sulfoxid | I | 1 | 1 | 2.5 | 2.5 |  |  |  |  | 5.33 |
| Acetamiprid | I | 16 | 2 | 2.0 | 42.7 | 10.2 (± 3.0) | 2.6 | 4.1 | 10.0 | 2.94 |
| Fluroxypyr-methyl | H | 2 | 1 | 47.7 | 242.5 | - | - | - | - | 2.42 |
| Fluopyram | F | 23 | 3 | 2.1 | 134.3 | 34.7 (± 7.9) | 12.1 | 18.7 | 40.3 | 1.31 |
| Tebuconazole | F | 47 | 5 | 2.1 | 97.7 | 21.3 (± 2.9) | 5.1 | 13.3 | 33.4 | 1.18 |
| Prothioconazole-desthio | F | 75 | 5 | 2.2 | 78.6 | 13.4 (± 1.6) | 5.0 | 8.7 | 14.8 | 1.11 |
| Tau-Fluvalinate | I | 2 | 2 | 8.4 | 10.0 | - | - | - | - | 0.80 |
| Pendimethalin | H | 26 | 5 | 2.1 | 43.4 | 8.8 (± 1.6) | 4.2 | 6.3 | 9.8 | 0.43 |
| Triadimenol | F | 23 | 5 | 2.2 | 80.8 | 21.2 (± 4.7) | 5.9 | 12.0 | 21.2 | 0.36 |
| Terbuthylazine | H | 10 | 3 | 2.1 | 5.4 | 3.3 (± 0.3) | 2.6 | 3.0 | 3.8 | 0.24 |
| Epoxiconazole | F | 11 | 2 | 2.0 | 16.4 | 5.4 (± 1.2) | 3.2 | 4.0 | 6.5 | 0.20 |
| Fenpropimorph | F | 9 | 4 | 2.2 | 17.7 | 5.8 (± 1.6) | - | 4.1 | - | 0.19 |
| Isoproturon | H | 11 | 3 | 2.4 | 23.3 | 9.5 (± 2.2) | 2.7 | 8.5 | 12.6 | 0.12 |
| Metconazole | F | 4 | 2 | 2.2 | 10.4 | 4.7 (± 1.7) | - | 3.1 | - | 0.12 |
| Flonicamid | I | 1 | 1 | 7.3 | 7.3 | - | - | - | - | 0.12 |
| Kresoxim-methyl | F | 2 | 1 | 2.1 | 12.0 | - | - | - | - | 0.11 |
| Prosulfocarb | H | 14 | 4 | 2.1 | 9.9 | 3.6 (± 0.6) | 2.3 | 2.5 | 3.9 | 0.10 |
| Dimethenamid-P | H | 8 | 4 | 3.6 | 11.8 | 5.7 (± 0.9) | - | 4.7 | - | 0.10 |
| Metalaxyl-M | F | 1 | 1 | 7.7 | 7.7 | - | - | - | - | 0.08 |
| Fuberidazole | F | 4 | 1 | 5.0 | 12.9 | 10.3 (± 1.6) | - | 11.6 | - | 0.07 |
| Metamitron | H | 1 | 1 | 6.6 | 6.6 | - | - | - | - | 0.07 |
| Metolachlor | H | 4 | 1 | 2.2 | 6.2 | 3.9 (± 0.8) | - | 3.5 | - | 0.06 |
| Cyprodinil | F | 4 | 1 | 2.1 | 5.2 | 3.6 (± 0.6) | - | 3.6 | - | 0.05 |
| Pyraclostrobin | F | 1 | 1 | 3.6 | 3.6 | - | - | - | - | 0.05 |
| Metrafenone | F | 4 | 3 | 3.0 | 4.8 | 3.9 (± 0.3) | - | 3.9 | - | 0.04 |
| Difenoconazole | F | 1 | 1 | 6.6 | 6.6 | - | - | - | - | 0.04 |
| Pencycuron | F | 1 | 1 | 3.3 | 3.3 | - | - | - | - | 0.03 |
| Spiroxamine | F | 2 | 2 | 2.2 | 2.5 | - | - | - | - | 0.02 |
| Trifloxystrobin | F | 2 | 2 | 2.2 | 4.4 | - | - | - | - | 0.02 |
| Picaridin | IR | 36 | 2 | 2.1 | 412.0 | 64.3 (± 15.8) | 4.0 | 18.0 | 89.4 |  |

1. F = fungicide, H = herbicide, I = insecticide, IR = insect repellent
   ^b)^ Lowest detected concentration according to LOD as obtained by LUFA Speyer, for better comparability of all sites.

**S3 Table C. Site specific summary.** Overview of 58 different substances detected in 56 pollen load samples at the “fruit” site during the 2012-2014 observation period, assorted according their respective maximum PHQ value.

| Substance | Pesticide class ^a)^ | Number of positive detections in 3 years | Number of years detected (out of 3 years) | Lowest detected conc. (µg/kg) ^b)^ | Highest detected conc. (µg/kg) | Mean (± ) SEM | 1. quartile | Median | 2. quartile | PHQ_max_ |
| --- | --- | --- | --- | --- | --- | --- | --- | --- | --- | --- |
| Clothianidin | I | 3 | 2 | 2.0 | 2.4 | 2.1 (± 0.1) | - | - | - | 600 (3x 500+) |
| Imidacloprid | I | 1 | 1 | 2.1 | 2.1 | - | - | - | - | 567.57 (1x 500+) |
| Fluazifop-butyl | H | 21 | 3 | 12.3 | 6,831.3 | 1,191.1 (± 457.9) | 32.2 | 72.9 | 391.8 | 108.43 (4x 50+) |
| Dimethomorph | F | 15 | 3 | 2.1 | 2,678.4 | 471.6 (± 224. 6) | 22.7 | 88.3 | 275.5 | 82.67 (2x 50+) |
| Indoxacarb | I | 5 | 3 | 4.0 | 20.0 | 7.6 (± 2.8) |  | 4.7 |  | 76.92 (1x) |
| Fenhexamid | F | 18 | 3 | 6.8 | 7,177.7 | 665.2 (± 411.4) | 18.9 | 63.3 | 175.1 | 70.32 (1x) |
| Nicotine | I | 1 | 1 | 3.0 | 3.0 | - | - | - | - | 37.5 |
| Etofenprox | I | 6 | 2 | 2.5 | 7.8 | 4.9 (± 0.7) | 3.4 | 5.0 | 6.1 | 28.89 |
| Thiacloprid | I | 46 | 3 | 3.5 | 470.4 | 89.2 (± 16.7) | 9.3 | 34.1 | 126.8 | 27.16 |
| Dimethoate | I | 1 | 1 | 2.8 | 2.8 | - | - | - | - | 23.33 |
| Azoxystrobin | F | 18 | 3 | 2.1 | 560.5 | 42.7 (± 29.9) | 3.1 | 3.8 | 9.6 | 22.42 |
| Fludioxonil | F | 32 | 3 | 5.2 | 1,085.1 | 113.8 (± 37.4) | 11.9 | 24.1 | 91.5 | 10.85 |
| Methiocarb | I | 1 | 1 | 3.1 | 3.1 | - | - | - | - | 6.60 |
| Tebuconazole | F | 28 | 3 | 2.0 | 484.5 | 69.3 (± 25.2) | - | 9.1 | - | 5.83 |
| Iprovalicarb | F | 11 | 3 | 3.4 | 974.7 | 131.6 (± 85.2) | - | 13.2 | - | 4.90 |
| Boscalid | F | 36 | 3 | 2.3 | 404.6 | 62.1 (± 14.6) | 7.1 | 17.0 | 86.9 | 4.05 |
| Myclobutanil | F | 15 | 3 | 2.2 | 136.8 | 32.4 (± 10.0) | 5.4 | 17.3 | 45.3 | 4.04 |
| Pirimicarb | I | 2 | 2 | 11.5 | 14.7 | - | - | - | - | 3.68 |
| Flusilazole | F | 2 | 1 | 19.8 | 115.6 | - | - | - | - | 3.42 |
| MCPA | H | 1 | 1 | 667 | 667.0 | - | - | - | - | 3.34 |
| Metrafenone | F | 31 | 3 | 2.1 | 368.5 | 62.3 (± 17.3) | 4.3 | 16.6 | 63.6 | 3.23 |
| Dimoxystrobin | F | 9 | 1 | 4.5 | 223.2 | 40.0 (± 21.9) | 7.2 | 15.8 | 35.1 | 2.81 |
| Epoxiconazole | F | 8 | 3 | 2.0 | 170.4 | 23.9 (± 19.6) | 2.4 | 2.8 | 4.1 | 2.05 |
| Pyraclostrobin | F | 13 | 3 | 2.5 | 124.0 | 51.4 (± 11.8) | 9.4 | 47.5 | 73.1 | 1.72 |
| Acetamiprid | I | 4 | 1 | 2.8 | 23.0 | 11.7 (±3.9) | - | 10.4 | - | 1.58 |
| Spiroxamine | F | 7 | 3 | 2.1 | 132.4 | 25.9 (± 16.9) | 2.6 | 3.0 | 19.2 | 1.32 |
| Metconazole | F | 8 | 2 | 2.4 | 94.4 | 21. 9 (± 10.3) | 6.9 | 8.8 | 17.1 | 1.11 |
| Trifloxystrobin | F | 24 | 3 | 2.1 | 218.3 | 32.3 (± 10.3) | 6.0 | 14.0 | 22.9 | 1.09 |
| Pirimicarb-desmethyl | I | 1 | 1 | 4.1 | 4.1 | - | - | - | - | 1.03 |
| Gamma-, Lambda-Cyhalothrin | I | 2 | 1 | 2.0 | 2.5 | - | - | - | - | 0.98 |
| Kresoxim-methyl | F | 8 | 2 | 2.6 | 106.3 | 37.1 (± 13.5) | 11.5 | 18.4 | 48.3 | 0.97 |
| Proquinazid | F | 13 | 3 | 2.4 | 110.2 | 27.6 (± 8.1) | 8.0 | 12.6 | 39.2 | 0.88 |
| Difenoconazole | F | 10 | 3 | 2.3 | 147.7 | 41.8 (± 15.5) | 3.7 | 9.7 | 74.6 | 0.83 |
| Coumaphos | V | 1 | 1 | 3.5 | 3.5 | - | - | - | - | 0.76 |
| Mandipropamid | F | 8 | 2 | 2.0 | 101.8 | 20.8 (±11.3) | 2.8 | 6.8 | 16.9 | 0.51 |
| Penconazole | F | 18 | 3 | 2.1 | 35.0 | 9.1 (± 2.0) | 4.0 | 6.1 | 9.0 | 0.31 |
| Quinoxyfen | F | 10 | 2 | 7.4 | 261.4 | 78.8 (± 24.8) | 18.2 | 46.6 | 114.6 | 0.26 |
| Tebufenozide | I | 5 | 2 | 2.0 | 24.8 | 11.1 (± 3.8) | - | 11.2 | - | 0.25 |
| Prosulfocarb | H | 22 | 3 | 2.0 | 24.2 | 6.2 (± 1.0) | 3.2 | 4.8 | 8.0 | 0.23 |
| Pendimethalin | H | 6 | 3 | 3.5 | 22.5 | 8.4 (± 2.7) | 4.2 | 5.5 | 8.4 | 0.22 |
| Cyprodinil | F | 27 | 3 | 2.0 | 1,282.6 | 117.4 (± 47.8) | 9.0 | 30.5 | 119.2 | 0.22 |
| Fluopicolide | F | 8 | 3 | 2.9 | 50.5 | 23.2 (± 6.5) | 7.1 | 19.0 | 35.2 | 0.21 |
| Cyflufenamid | F | 8 | 2 | 3.2 | 20.1 | 8.6 (± 2.3) | 3.3 | 4.7 | 13.4 | 0.20 |
| Metalaxyl-M | F | 8 | 2 | 2.6 | 17.6 | 6.3 (± 1.6) | 3.8 | 5.1 | 6.0 | 0.18 |
| Chlorantraniliprole | I | 1 | 1 | 18.1 | 18.1 | - | - | - | - | 0.17 |
| Prothioconazole-desthio | F | 12 | 2 | 2.0 | 11.5 | 4.6 (± 0.8) | 2.6 | 3.9 | 4.8 | 0.16 |
| Methoxyfenozide | I | 4 | 2 | 6.3 | 14.4 | 8.9 (± 1.6) | - | 7.5 | - | 0.14 |
| Thymol | V | 1 | 1 | 8.0 | 8.0 | - | - | - | - | 0.14 |
| Propamocarb | F | 2 | 1 | 2.8 | 8.0 | - | - | - | - | 0.10 |
| Benthiavalicarb isopropyl | F | 2 | 1 | 4.4 | 8.9 | - | - | - | - | 0.09 |
| Zoxamide | F | 1 | 1 | 6.6 | 6.6 | - | - | - | - | 0.07 |
| Isoproturon | H | 4 | 2 | 2.2 | 9.5 | 4.4 (± 1.5) | - | 3.1 | - | 0.05 |
| Fenpropimorph | F | 3 | 2 | 2.8 | 3.6 | 3.1 (± 0.2) | - | - | - | 0.04 |
| Fenoxycarb | I | 1 | 1 | 6.3 | 6.3 | - | - | - | - | 0.03 |
| Cyproconazole | F | 1 | 1 | 27.7 | 27.7 | - | - | - | - | 0.03 |
| Pyrimethanil | F | 1 | 1 | 2.6 | 2.6 | - | - | - | - | 0.03 |
| Picoxystrobin | F | 1 | 1 | 4.2 | 4.2 | - | - | - | - | 0.02 |
| Famoxadone | F | 1 | 1 | 2.8 | 2.8 | - | - | - | - | 0.003 |

1. F = fungicide, H = herbicide, I = insecticide, IR = insect repellent V = varroacide
2. Lowest detected concentration according to LOD as obtained by LUFA Speyer, for better comparability of all sites.
