# Supplementary material for: Pesticide residue survey of pollen loads collected by honeybees (Apis mellifera) in daily intervals at three agricultural sites in South Germany
Source: PLoS One. 2018 Jul 6;13(7):e0199995. doi: 10.1371/journal.pone.0199995 (PMC6034819; doi:10.1371/journal.pone.0199995)
Supplement: S5 Tables — A-I. In depth residue analysis of single days. Pollen pellets of composite samples were sorted according to pellet color into sub-fractions. Pesticide concentrations are given for the composite sample, as well as for each fraction (μg/kg). For each fraction, the dominating plant species is given. (DOCX) [file pone.0199995.s005.docx]

**S5 Table A. In depth residue analysis of single days.** Pollen pellets of composite sample 27.05.2013 of site “grain” were sorted according to pellet color into five sub-fractions. Pesticide concentrations are given for the composite sample, as well as for each fraction (µg/kg). For each fraction, the dominating plant species is given. Concentrations of the composite sample are given according to the LOQ values as obtained by the JKI analysis. Fractions were analyzed by LUFA Speyer.

| Pesticides | Pesticide class | Composite sample | *Brassica* sp. | *Acer* L. | *Pyrus* group | *Salix* ssp. | *Ranunculus* L. |
| --- | --- | --- | --- | --- | --- | --- | --- |
| Boscalid | F | 12.5 | 29.2 | 3.1 | 3.0 |  | 5. 7 |
| Dimoxystrobin | F | 7.6 | 6.9 |  |  |  | 5.0 |
| Fluopyram | F | 131.5 | 288.0 | 10.9 | 6.8 | 4.8 | 2.2 |
| Pendimethalin | H |  |  | 3.9 |  |  |  |
| Prosulfocarb | H | 2.2 |  | 3.6 | 2.9 |  | 3.7 |
| Prothioconazole-desthio | F | 25.0 | 30.9 | 12.0 | 3.8 | 7. 7 | 9.7 |
| Spiroxamine | F |  |  |  |  |  | 3.8 |
| Tebuconazole | F |  | 8.4 |  |  |  |  |
| Tebufenozid | I |  |  |  | 5.0 |  |  |
| Thiacloprid | I | 61.8 | 186.5 | 6.3 | 7.4 |  | 14.8 |
| Triadimenol | F |  | 14.6 |  |  |  |  |

F = fungicide, I = insecticide, H = herbicide

**S5 Table B. In depth residue analysis of single days.** Pollen pellets of composite sample 06.06.2013 of site “fruit” were sorted according to pellet color into twelve sub-fractions. Pesticide concentrations are given for the composite sample, as well as for each fraction (µg/kg). For each fraction, the dominating plant species is given. Concentrations of the composite sample are given according to the LOQ values as obtained by the JKI analysis. Fractions were analyzed by LUFA Speyer.

| Pesticides | Pesticide class | Composite sample | *Poaceae*/*Brassica* sp. | *Caryo-phyllaceae* Juss. | *Taraxacum* F.H. Wigg. | *Brassica* sp./ *Sambucus ssp.* | *Poaceae* | *Fragaria* L. | *Robinia pseudoacacia* L. | *Galium* L. | *Rubus* group | *Knautia avensis* | *Trifolium pratense / Pinus* L. | *Pinus* L. |
| --- | --- | --- | --- | --- | --- | --- | --- | --- | --- | --- | --- | --- | --- | --- |
| Azoxystrobin | F | 560.5 | 56.3 | 32.6 | 27.5 | 19.8 | 48.7 | 567.8 | 29.5 | 22.3 | 35.7 | 83.7 | 35.4 | 53.9 |
| Boscalid | F | 7.1 | 3.7 |  | 1.7 | 2.3 | 4.5 | 207.5 | 76.9 | 7.0 | 1.9 |  | 7.2 |  |
| Carbendazim | F | 0.5 |  |  |  |  |  |  |  |  |  |  |  |  |
| Clothianidin | I | 0.3 |  |  |  |  |  |  |  |  |  |  |  |  |
| Cyprodinil | F | 20.1 | 4.3 |  | 2.6 | 2.6 | 4.2 | 452.3 | 3.0 |  | 27.2 |  | 3.1 |  |
| Dimethenamid-P | H | 0.5 |  |  | 1.6 | 1.9 |  | 3.1 |  | 1.7 | 2.3 |  | 2.4 |  |
| Dimethoate | I | 0.4 |  |  |  |  |  |  |  | 2.4 |  |  |  |  |
| Dimethomorph | F |  |  |  |  |  |  |  |  |  |  |  |  | 12.6 |
| Epoxiconazole^a^ | F | 2.4 |  |  |  |  |  |  |  |  |  |  |  |  |
| Fenhexamid | F | 7,177.7 | 115.3 | 55.7 | 64.6 | 63.0 | 121.3 | 4,452.4 | 38.8 | 24.7 | 28.1 | 32.1 | 33.3 | 14.9 |
| Fenoxycarb | I |  |  |  |  |  |  |  | 2.6 |  |  |  |  |  |
| Fenpropimorph | F | 3.6 | 3.1 |  | 2.0 |  | 4.4 |  |  | 279.8 | 2.4 |  |  |  |
| Fenpyroximate | I | 1.1 |  | 4.2 |  |  |  |  |  |  | 5.1 | 4.5 |  |  |
| Fluazifop^a^ | H | 1.7 |  |  |  |  |  |  |  |  |  |  |  |  |
| Fludioxonil^a^ | F | 45.2 |  |  |  |  |  |  |  |  |  |  |  |  |
| Flufenoxuron | I |  |  |  |  |  |  |  |  |  | 3.3 |  |  |  |
| Imidacloprid | I |  | 1.9 |  |  |  |  |  |  |  |  |  |  |  |
| Kresoxim-Methyl | F |  |  |  |  |  |  | 7.9 |  | 11.0 |  |  |  |  |
| Metrafenone | F | 7.2 | 12.4 | 2.1 | 19.0 | 11.2 | 11.2 | 22.9 | 72.7 | 26.9 | 21.3 | 2.0 | 22.1 | 8.2 |
| Myclobutanil | F |  |  |  |  |  |  | 64.9 |  |  |  |  |  |  |
| Penconazole | F | 13.1 |  |  |  |  |  | 233.6 |  |  |  |  |  |  |
| Pendimethalin | H |  |  |  |  |  |  | 4.7 |  |  |  |  |  |  |
| Pirimicarb | I |  |  |  |  |  |  |  |  |  | 3.2 | 14.7 | 2.4 |  |
| Proquinazid | F | 0.4 |  |  |  |  |  |  |  |  |  |  |  |  |
| Prosulfocarb | H |  | 5.1 |  | 3.0 | 2.2 | 4.4 |  |  | 3.1 | 4.5 | 3.7 | 3.6 | 3.9 |
| Prothioconazole-desthio | F |  | 2.9 |  | 3.0 | 3.4 | 2.5 | 11.5 |  | 8.2 | 9 |  |  |  |
| Pyraclostrobin | F | 1.3 |  |  |  |  |  | 17.7 | 17.3 |  |  |  |  |  |
| Spiroxamine | F | 3.0 |  |  |  |  | 3.3 |  |  |  | 4.2 |  |  |  |
| Tau-Fluvalinate | I |  |  |  |  |  |  |  |  |  | 14.7 | 18.9 |  |  |
| Tebuconazole | F | 421.1 | 22.5 | 2,589.3 | 15.4 | 9.0 | 25.8 | 16.1 | 15.2 | 13.3 |  |  | 10.6 | 14.0 |
| Tebufenozid | I |  | 2.3 |  |  |  | 2.4 |  |  |  |  |  |  |  |
| Thiacloprid | I | 14.0 | 3.1 | 3.7 |  | 4.1 | 2.3 | 88.2 | 44.0 | 4.7 | 4.0 |  | 4.2 | 6.3 |
| Thiacloprid-amid^a^ | I | 0.6 |  |  |  |  |  |  |  |  |  |  |  |  |
| Trifloxystrobin | F | 135.9 | 18.9 | 17.3 | 21.7 | 15.3 | 23.8 | 589.4 | 21.5 | 17.3 | 32.1 | 47.0 | 32.1 | 8.6 |

F = fungicide, I = insecticide, H = herbicide
^a^substances have not been included in this analysis at LUFA Speyer.

**S5 Table C. In depth residue analysis of single days.** Pollen pellets of composite sample 01/02.07.2013 of site “fruit” were sorted according to pellet color into five sub-fractions. Pesticide concentrations are given for the composite sample, as well as for each fraction (µg/kg). For each fraction, the dominating plant species is given. Concentrations of the composite sample are given according to the LOQ values as obtained by the JKI analysis. Fractions were analyzed by LUFA Speyer.

| Pesticides | Pesticide class | Composite sample | *Vitis vinifera* L. | *Rubus* group | *Castanea sativa* Mill. | *Plantago* L. | *Achillea* L. |
| --- | --- | --- | --- | --- | --- | --- | --- |
| Azoxystrobin | F | 0.4 |  | 20.8 |  |  |  |
| Benthiavalicarb-P-isopropyl | F | 0.4 |  |  |  |  |  |
| Boscalid | F | 178.9 | 395.1 | 19.2 | 44.4 | 12.5 | 22.2 |
| Cyflufenamid | F | 17.1 | 25.9 | 4.9 | 5.9 | 16.8 | 5.5 |
| Cyprodinil | F | 2.0 |  | 13.1 | 5.0 |  |  |
| Deltamethrin^a^ | I | 1.0 |  |  |  |  |  |
| Difenoconazole | F | 147.7 | 145.1 | 7.4 | 6.4 | 3.4 |  |
| Dimethenamid-P | H |  |  |  |  |  | 2.8 |
| Dimethoate | I | 0.7 |  |  |  |  |  |
| Dimethomorph | F | 2,567.2 | 2,638.4 | 173.1 | 379.0 | 69.4 | 170.9 |
| Epoxiconazole^a^ | F | 3.2 |  |  |  |  |  |
| Fenhexamid | F |  |  | 2.9 |  |  |  |
| Fludioxonil^a^ | F | 1.5 |  |  |  |  |  |
| Fluopicolide | F | 30.2 | 122.3 | 7.0 | 86.1 | 4.9 | 11.8 |
| Fluopyram^b^ | F |  | 1,104.7 | 77.2 | 108.5 | 59.0 | 77.3 |
| Iprovalicarb | F | 974.7 | 639.1 | 62.9 | 469.0 | 24.8 | 25.0 |
| Isoxaben | H | 0.2 |  |  |  |  |  |
| Kresoxim-methyl | F | 106.3 | 200.0 | 24.2 | 39.3 | 25.1 | 25.9 |
| Mandipropamid | F | 11.9 | 60.6 | 3.8 | 7.4 |  |  |
| Metalaxyl-M | F | 17.6 | 52.8 | 5.2 | 4.5 | 3.5 | 2.2 |
| Metrafenone | F | 268.5 | 538.0 | 45.0 | 75.3 | 33.4 | 26.7 |
| Myclobutanil | F | 19.0 | 40.4 | 3.9 | 2.1 |  |  |
| Penconazole | F | 9.0 | 6.1 |  | 5.5 | 32.7 |  |
| Pendimethalin | H |  |  |  |  | 2.5 |  |
| Proquinazid | F | 53.1 |  |  |  |  |  |
| Prosulfocarb | H |  |  |  | 3.0 |  | 4.0 |
| Prothioconazole-desthio | F | 6.2 | 5.2 |  |  |  |  |
| Pyraclostrobin | F | 18.0 | 23.0 |  |  | 3.4 |  |
| Quinoxyfen | F | 38.2 |  |  |  |  |  |
| Spiroxamine | F | 0.2 |  |  |  |  |  |
| Tebuconazole | F | 484.5 | 977.2 | 61.6 | 126.8 | 22.3 | 30.8 |
| Thiacloprid | I | 3.8 |  | 9.1 | 4.0 | 7.0 |  |

F = fungicide, I = insecticide, H = herbicide
^a^substances have not been included in this analysis at LUFA Speyer.
^b^substance has not been included in this analysis at JKI Berlin.

**S5 Table D. In depth residue analysis of single days.** Pollen pellets of composite sample 14/15.05.2014 of site “fruit” were sorted according to pellet color into eight sub-fractions. Pesticide concentrations are given for the composite sample, as well as for each fraction (µg/kg). For each fraction, the dominating plant species is given. Concentrations of the composite sample are given according to the LOQ values as obtained by the JKI analysis. Fractions were analyzed by LUFA Speyer.

| Pesticides | Pesticide class | Composite sample | *Cornus* L. | *Acer* L. | *Brassica* sp. | *Fragaria* L. / *Pyracantha* M. Roem. | *Acer* L. / *Veronica* | *Ranunculus* L. | *Aesculus hippocastanum* L. | *Rubus* group |
| --- | --- | --- | --- | --- | --- | --- | --- | --- | --- | --- |
| Azoxystrobin | F | 42.5 | 3.2 | 2.0 | 12.0 | 996.8 | 3.0 | 4.7 |  | 4.8 |
| Boscalid | F | 5.5 |  | 13.9 | 1.6 | 87.2 |  |  |  |  |
| Clothianidin | I | 0.5 |  |  |  |  |  |  |  |  |
| Cyprodinil | F | 1,282.6 | 49.4 | 48.5 | 60.3 | 19,854.8 | 63.9 | 62.9 | 60.5 |  |
| Dimethenamid-P | H | 0.9 | 2.3 |  | 4.4 | 11.2 |  |  |  |  |
| Dimethoate | I | 0.2 |  |  |  |  |  |  |  |  |
| Fenhexamid | F | 302.1 | 4.1 | 2.3 |  | 207.8 |  |  |  |  |
| Fenpyroximate | I |  |  |  |  |  |  |  | 2.0 |  |
| Fluazifop | H | 38.0 |  |  |  |  |  |  |  |  |
| Fludioxonil^a^ | F | 1,085.1 |  |  |  |  |  |  |  |  |
| Indoxacarb | I | 4.0 |  |  |  |  |  |  | 13.4 |  |
| Metconazole | F | 2.4 |  |  |  |  |  |  |  |  |
| Metrafenone | F | 5.9 | 16.0 | 6.2 | 6.5 | 26.5 |  | 2.5 | 15.4 | 9.3 |
| Pendimethalin | H | 3.0 | 3.2 |  | 4.0 | 18.7 |  |  |  |  |
| Pirimicarb | I |  |  |  |  | 2.1 |  | 3.1 | 3.0 | 14.1 |
| Proquinazid | I | 1.2 |  |  |  |  |  |  |  |  |
| Prosulfocarb | H | 3.0 | 10.8 | 4.6 | 4.4 | 8.6 | 3.2 | 7.2 | 5.0 | 4.8 |
| Pyraclostrobin | F | 2.5 |  | 2.4 |  | 15.0 |  |  |  |  |
| Spiroxamine | F | 0.4 |  |  |  |  |  |  |  |  |
| Tebuconazole | F | 1.3 |  |  | 6.5 |  |  |  |  |  |
| Thiacloprid | I | 8.6 | 2.3 | 2.1 | 9.3 | 4.0 | 4.8 | 28.5 | 4.1 |  |
| Thiacloprid-amid^a^ | I | 0.6 |  |  |  |  |  |  |  |  |
| Trifloxystrobin | F | 7.8 |  |  |  |  |  |  | 17.8 | 3.4 |

F = fungicide, I = insecticide, H = herbicide

^a^substances have not been included in this analysis at LUFA Speyer.

**S5 Table E. In depth residue analysis of single days.** Pollen pellets of composite sample 03.05.2012 of site “fruit” were sorted according to pellet color into ten sub-fractions. Pesticide concentrations are given for the composite sample, as well as for each fraction (µg/kg). For each fraction, the dominating plant species is given. Concentrations of the composite sample are given according to the LOQ values as obtained by the JKI analysis. Fractions were analyzed by LUFA Speyer.

| Pesticides | Pesticide class | Composite sample | *Brassica* sp. | *Taraxacum* F.H. Wigg. | *Pyrus group* | *Pyrus group* | Unknown^b^ | unknown^b^ | unknown^b^ | unknown^b^ | unknown^b^ | unknown^b^ |
| --- | --- | --- | --- | --- | --- | --- | --- | --- | --- | --- | --- | --- |
| Acephat | I |  |  |  |  |  |  |  |  | 2.4 |  |  |
| Aldoxycarb | I |  |  |  |  |  |  |  |  |  | 5.9 | 4.7 |
| Azoxystrobin | F | 3.9 | 3.9 |  |  |  |  |  |  |  |  |  |
| Boscalid | F | 404.6 | 2,370.1 | 23.0 | 19.3 | 23.3 | 570.9 | 8.2 |  | 38.2 |  |  |
| Clothianidin | I | 0.9 |  |  |  |  |  |  |  |  |  |  |
| Cyprodinil | F | 4.2 |  |  |  |  | 105.6 |  | 398.5 |  |  |  |
| Dimethoate | I | 0.3 |  |  |  |  |  |  |  |  |  |  |
| Dimoxystrobin | F | 223.2 | 534.0 | 8.7 | 4.8 | 16.8 | 7.6 | 5.9 | 10.3 | 19.3 |  |  |
| Ethofumesate | H |  |  |  | 32.4 | 64.5 |  |  |  |  |  |  |
| Fenhexamid | F | 32.9 |  |  | 21.5 |  | 2,071.2 |  |  |  |  |  |
| Fenpropidin | F | 0.5 |  |  |  |  |  |  |  |  |  |  |
| Fluazifop | H | 94.3 | 169.3 | 5.5 |  |  | 255.8 | 10.6 | 5.1 |  |  | 16.1 |
| Fludioxonil | F | 12.7 |  |  | 7.2 |  | 406.5 |  | 1,013.1 | 6.5 |  |  |
| Gamma-, Lambda-Cyhalothrin^a^ | I | 0.7 |  |  |  |  |  |  |  |  |  |  |
| Isoxaben | H |  |  |  |  |  | 14.3 |  |  |  |  |  |
| MCPA | H |  |  |  |  |  |  |  |  |  |  | 10.9 |
| Mecoprop | H |  |  |  |  |  |  |  |  |  |  | 6.5 |
| Methiocarb | I | 0.7 |  |  |  |  |  |  |  |  |  |  |
| Methoxyfenozide | I | 7.1 |  | 5.0 | 51.3 | 2.5 |  | 34.7 | 30.7 |  |  |  |
| Pendimethalin | H |  | 3.4 |  |  |  | 5.8 |  |  |  |  |  |
| Phosalone^a^ | I | 0.6 |  |  |  |  |  |  |  |  |  |  |
| Propamocarb | F | 1.9 |  |  |  |  |  |  |  |  |  |  |
| Propyzamide | H | 0.3 |  |  |  |  | 22.2 |  |  |  |  |  |
| Pyraclostrobin | F | 2.6 |  |  |  |  | 116.0 |  |  |  |  |  |
| Spiroxamine | F | 2.3 |  |  |  |  |  |  |  |  |  |  |
| Tebuconazole | F | 5.5 |  |  |  |  |  |  |  |  |  |  |
| Tebufenozid | I | 14.8 |  | 4.4 | 39.6 | 3.1 |  | 36.4 |  |  |  |  |
| Tebufenpyrad | I |  | 4.1 |  |  |  |  |  |  |  |  |  |
| Thiacloprid | I | 318.2 | 1,107.7 | 18.4 | 3.5 | 19.1 | 115.9 |  | 147.7 | 15.8 | 23.2 |  |
| Trifloxystrobin | F | 17.6 |  | 9.6 | 73.9 | 9.5 | 493.3 | 34.1 |  | 3.1 |  |  |

F = fungicide, I = insecticide, H = herbicide
^a^substances have not been included in this analysis at LUFA Speyer.
^b^unknown plant species: due to problems in the laboratory, not enough pollen was left over for palynological analysis in this sub-fraction

**S5 Table F. In depth residue analysis of single days.** Pollen pellets of composite sample 18.05.2012 of site “fruit” were sorted according to pellet color into six sub-fractions. Pesticide concentrations are given for the composite sample, as well as for each fraction (µg/kg). For each fraction, the dominating plant species is given. Concentrations of the composite sample are given according to the LOQ values as obtained by the JKI analysis. Fractions were analyzed by LUFA Speyer.

| Pesticides | Pesticide class | Composite sample | *Brassica* sp. | unknown^b^ | unknown^b^ | unknown^b^ | unknown^b^ | unknown^b^ |
| --- | --- | --- | --- | --- | --- | --- | --- | --- |
| Acephat | I |  |  |  |  | 2.2 | 2.0 |  |
| Aldoxycarb | I |  |  |  |  |  |  | 2.3 |
| Azoxystrobin | F | 0.3 |  | 18.0 |  |  |  |  |
| Boscalid | F | 42.0 | 102.3 | 84.0 |  |  |  |  |
| Clothianidin | I | 2.0 |  |  |  |  |  |  |
| Cyprodinil | F |  |  | 364.8 |  |  |  |  |
| Dimoxystrobin | F | 23.9 | 21.5 |  | 3.6 |  |  |  |
| Etofenprox^a^ | I | 3.0 |  |  |  |  |  |  |
| Fenhexamid | F | 3.0 |  | 113.4 |  |  |  |  |
| Fenoxycarb | I | 6.3 | 2.1 | 836.2 |  |  |  |  |
| Fluazifop | H | 13.8 | 11.8 |  |  |  | 6.5 |  |
| Fludioxonil | F | 13.6 | 8.2 | 773.2 | 10.0 |  |  | 5.2 |
| Ioxynil | H |  |  |  |  |  | 9.0 |  |
| MCPA | H |  |  |  | 6.4 |  |  |  |
| Mecoprop | H |  |  |  |  | 5.1 | 8.3 |  |
| Metrafenone | F | 2.1 |  | 4.2 |  |  |  |  |
| Penconazole | F | 1.4 |  | 25.6 |  |  |  |  |
| Pendimethalin | H |  |  | 3.3 |  |  |  |  |
| Pirimicarb | I | 0.9 |  | 27.4 |  |  |  |  |
| Proquinazid | I | 1.6 |  |  |  | 110.8 |  |  |
| Pyraclostrobin | F | 0.2 |  | 8.5 |  |  |  |  |
| Quinoxyfen | F | 0.6 |  |  |  |  |  |  |
| Tebuconazole | F | 2.9 |  |  |  |  |  |  |
| Thiacloprid | I | 390.3 | 267.4 | 34.2 |  |  |  |  |
| Trifloxystrobin | F | 0.6 |  | 8.5 |  |  | 3.9 |  |

F = fungicide, I = insecticide, H = herbicide
^a^substances have not been included in this analysis at LUFA Speyer.
^b^unknown plant species: due to problems in the laboratory, not enough pollen was left over for palynological analysis in this sub-fraction

**S5 Table G. In depth residue analysis of single days.** Pollen pellets of composite sample 25.05.2012 of site “fruit” were sorted according to pellet color into ten sub-fractions. Pesticide concentrations are given for the composite sample, as well as for each fraction (µg/kg). For each fraction, the dominating plant species is given. Concentrations of the composite sample are given according to the LOQ values as obtained by the JKI analysis. Fractions were analyzed by LUFA Speyer.

| Pesticides | Pesticide class | Composite sample | *Sinapis* sp. | *Cornus* L. | *Phacelia tanacetifolia* Benth. | unknown^b^ | unknown^b^ | unknown^b^ | unknown^b^ | unknown^b^ | unknown^b^ | unknown^b^ |
| --- | --- | --- | --- | --- | --- | --- | --- | --- | --- | --- | --- | --- |
| Acephat | I |  |  |  |  | 2.5 |  |  |  |  |  |  |
| Azoxystrobin | F | 0.4 |  |  |  |  |  | 56.9 |  |  |  |  |
| Boscalid | F | 7.1 | 7.3 |  |  |  | 120.7 | 89.2 |  |  |  |  |
| Cyprodinil | F | 30.5 |  |  |  |  |  | 2,062.1 |  |  |  |  |
| Dimethenamid-P | H | 0.3 |  |  |  |  |  |  |  |  |  |  |
| Dimethoate | I | 2.8 |  |  | 6.2 |  |  |  |  |  |  |  |
| Dimoxystrobin | F | 0.6 | 2.8 |  |  |  |  |  |  |  |  |  |
| Epoxiconazole^a^ | F | 2.3 |  |  |  |  |  |  |  |  |  |  |
| Fenoxycarb | I |  |  |  |  |  |  |  | 58.7 | 2.7 |  |  |
| Fenpyroximate | I |  |  |  |  |  | 9.8 |  | 4.2 |  |  |  |
| Fenhexamid | F |  |  |  |  |  |  | 185.0 |  |  |  |  |
| Fludioxonil | F | 312.3 | 6.1 | 25.0 | 5.1 |  | 78.0 | 3,182.2 | 61.4 | 73.0 | 24.1 |  |
| Ioxynil | H |  |  |  |  |  |  |  |  |  |  | 7.8 |
| Isoproturon | H |  |  |  |  |  | 71.7 |  |  |  |  |  |
| MCPA | H |  |  |  |  |  |  |  |  |  |  | 8.9 |
| Mecoprop | H |  |  |  |  |  |  |  |  |  |  | 18.3 |
| Metrafenone | F | 18.4 | 10.8 | 34.7 | 6.0 | 19.4 | 3.9 | 23.4 | 3.7 | 611.2 | 21.3 |  |
| Penconazole | F | 0.5 |  |  |  |  |  |  |  |  |  |  |
| Pendimethalin | H |  |  |  |  |  |  | 6.6 |  |  |  |  |
| Pirimicarb | I |  |  |  | 2.1 |  |  |  |  |  |  |  |
| Proquinazid | F | 0.3 |  |  |  |  |  | 3.5 |  |  |  |  |
| Pyraclostrobin | F | 0.2 |  |  |  |  | 10.3 | 9.3 | 6.7 |  |  |  |
| Quinoxyfen | F | 0.6 |  |  |  |  |  |  |  |  |  |  |
| Spiroxamine | F | 32.3 | 2.9 |  |  |  |  |  |  | 314.3 |  |  |
| Tebufenozid | I |  |  |  |  |  | 17.4 |  |  |  |  |  |
| Teflubenzuron | I |  |  |  | 2.2 |  |  |  |  |  |  |  |
| Terbuthylazine | H | 0.9 |  |  |  |  |  |  |  |  |  |  |
| Thiacloprid | I | 76.5 | 257.4 | 5.9 |  |  |  | 690.7 |  |  | 3.8 |  |
| Trifloxystrobin | F | 1.2 |  |  | 14.6 |  |  | 11.7 | 8.6 |  |  |  |

F = fungicide, I = insecticide, H = herbicide
^a^substances have not been included in this analysis at LUFA Speyer.
^b^unknown plant species: due to problems in the laboratory, not enough pollen was left over for palynological analysis in this sub-fraction

**S5 Table H. In depth residue analysis of single days.** Pollen pellets of composite sample 28.05.2012 of site “fruit” were sorted according to pellet color into twelve sub-fractions. Pesticide concentrations are given for the composite sample, as well as for each fraction (µg/kg). For each fraction, the dominating plant species is given. Concentrations of the composite sample are given according to the LOQ values as obtained by the JKI analysis. Fractions were analyzed by LUFA Speyer.

| Pesticides | Pesticide class | Composite sample | *Sinapis* sp. | *Achillea* L*.* | *Phacelia tanacetifolia* Benth. | unknown^b^ | unknown^b^ | unknown^b^ | unknown^b^ | unknown^b^ | unknown^b^ | unknown^b^ | unknown^b^ | unknown^b^ |
| --- | --- | --- | --- | --- | --- | --- | --- | --- | --- | --- | --- | --- | --- | --- |
| Acephat | I |  |  |  |  |  |  |  |  |  | 2.2 | 2.9 |  |  |
| Azoxystrobin | F | 77.7 |  | 1,275.5 | 2.9 | 4.7 | 10.6 | 4.2 | 2.3 |  |  |  | 5.1 | 12.4 |
| Boscalid | F | 11.4 |  | 2.2 |  |  | 5.7 |  | 17.2 |  |  | 155.3 |  |  |
| Cyproconazole^a^ | F | 27.7 |  |  |  |  |  |  |  |  |  |  |  |  |
| Cyprodinil | F | 140.5 |  | 1,395.7 |  |  |  |  |  |  |  | 1,063.1 |  |  |
| Dimethenamid-P | H | 0.8 |  |  |  |  |  |  |  |  |  |  |  |  |
| Dimethoate | I | 0.2 |  |  |  |  |  |  |  |  | 5.2 |  |  |  |
| Dimoxystrobin | F | 7.2 |  |  |  | 3.8 |  |  |  |  |  |  |  |  |
| Epoxiconazole^a^ | F | 170.4 |  |  |  |  |  |  |  |  |  |  |  |  |
| Ethofumesate | H |  |  |  |  |  |  |  |  |  | 14.5 |  |  |  |
| Fenazaquin | I |  |  |  |  | 2.1 |  |  |  |  |  |  |  |  |
| Fenoxycarb | I |  |  |  |  |  |  |  |  |  |  |  | 3.8 | 13.2 |
| Fenpropimorph | F |  |  | 3.4 |  |  |  |  |  |  |  |  |  |  |
| Fluazifop-butyl^a^ | H | 0.6 |  |  |  |  |  |  |  |  |  |  |  |  |
| Fludioxonil^a^ | F | 72.9 |  |  |  |  |  |  | 10.8 |  |  | 5,377.9 |  |  |
| Isoproturon | H |  |  |  |  |  |  |  |  |  | 16.7 |  |  |  |
| Kresoxim-Methyl | F |  |  | 2.4 |  |  |  |  |  |  |  |  |  |  |
| MCPA | H | 667.0 |  | 2,553.0 | 11.6 | 26.3 | 20.0 | 39.1 | 8.2 | 15.3 | 15.9 | 12.6 | 14.8 | 255.1 |
| Metconazol | F | 94.4 |  | 1,125.1 |  |  |  |  |  |  |  |  |  |  |
| Methoxyfenozide | I |  |  |  |  |  |  |  |  |  |  |  | 2.4 |  |
| Metolachlor | H |  |  | 21.5 |  |  |  |  |  |  |  |  |  |  |
| Metrafenone | F | 9.6 |  | 35.6 | 9.7 | 106.2 | 31.8 | 5.6 | 4.6 | 5.2 |  |  | 17.2 | 13.0 |
| Oxydemeton-methyl | I | 1.4 |  |  |  |  |  |  |  |  |  |  |  |  |
| Pendimethalin | H |  |  | 3.9 |  |  |  |  |  |  |  |  |  | 3.0 |
| Pyraclostrobin | F |  |  |  |  |  |  |  | 2.0 |  |  | 11.6 |  |  |
| Quinoxyfen | F | 0.7 |  |  |  |  |  |  |  |  |  |  |  |  |
| Spiroxamine | F | 1.0 |  | 4.2 |  |  |  |  |  |  |  |  |  |  |
| Thiacloprid | I | 76.6 | 169.2 |  |  |  |  |  |  |  |  | 739.8 |  |  |
| Trifloxystrobin | F | 1.4 |  | 7.0 | 5.5 |  | 4.1 |  |  |  |  | 16.3 |  | 2.3 |

F = fungicide, I = insecticide, H = herbicide
^a^substances have not been included in this analysis at LUFA Speyer.
^b^unknown plant species: due to problems in the laboratory, not enough pollen was left over for palynological analysis in this sub-fraction

**S5 Table I. In depth residue analysis of single days.** Pollen pellets of composite sample 14.06.2012 of site “fruit” were sorted according to pellet color into nine sub-fractions. Pesticide concentrations are given for the composite sample, as well as for each fraction (µg/kg). For each fraction, the dominating plant species is given. Concentrations of the composite sample are given according to the LOQ values as obtained by the JKI analysis. Fractions were analyzed by LUFA Speyer.

| Pesticides | Pesticide class | Composite sample | *Vitis vinifera* L. | *Phacelia tanacetifolia* Benth. | unknown^b^ | unknown^b^ | unknown^b^ | unknown^b^ | unknown^b^ | unknown^b^ | unknown^b^ |
| --- | --- | --- | --- | --- | --- | --- | --- | --- | --- | --- | --- |
| Aldoxycarb | I |  |  |  |  |  |  | 3.0 |  |  |  |
| Azoxystrobin | F | 26.0 | 101.9 | 3.8 | 421.2 | 8.1 | 6.8 |  | 25.9 |  | 7.9 |
| Benthiavalicarb-P-isopropyl | F | 4.4 |  |  |  |  |  |  |  |  |  |
| Boscalid | F | 200.7 | 396.3 | 5.2 | 509.8 | 207.2 | 11.8 |  |  | 15.2 | 45.5 |
| Dimethomorph | F | 841.7 | 3,747.7 | 70.6 | 3,567.3 | 926.8 | 48.3 |  | 37.4 | 101.6 | 116.8 |
| Dithianon^c^ | F |  | 65.5 |  | 72.0 |  |  |  |  |  |  |
| Epoxiconazole^a^ | F | 2.4 |  |  |  |  |  |  |  |  |  |
| Fenhexamid | F | 2.2 |  |  | 14.4 |  |  | 3,682.5 |  |  |  |
| Fenpyroximate | I |  | 2.0 |  |  |  |  |  |  |  |  |
| Fludioxonil^a^ | F | 2.7 |  |  | 5.8 |  |  | 168.0 |  |  |  |
| Fluopyram^c^ | F |  | 25.3 |  | 29.3 |  |  |  |  |  |  |
| Ioxynil | H |  |  |  |  |  |  | 5.0 |  |  |  |
| Iprovalicarb | F | 17.6 | 21.7 | 4.0 | 46.0 | 4.9 |  |  |  |  |  |
| Kresoxim-Methyl | F |  | 227.4 | 3.2 | 228.4 | 125.6 |  |  |  |  |  |
| MCPA | H |  | 5.6 |  |  |  |  | 28.4 | 6.7 | 5.0 |  |
| Mepronil | F |  | 4.5 |  |  |  |  |  |  |  |  |
| Metalaxyl-M | F | 5.1 | 8.4 |  |  |  |  |  |  |  |  |
| Metrafenone | F | 112.2 | 324.7 | 10.7 | 320.6 | 31.3 | 11.8 |  | 2.0 | 13.1 | 9.3 |
| Myclobutanil | F | 136.8 | 291.9 |  | 256.2 | 11.6 | 4.7 |  |  |  | 18.9 |
| Penconazole | F | 3.9 | 2.2 |  | 3.5 |  |  |  |  |  |  |
| Pendimethalin | H |  |  |  |  |  |  |  | 11.0 |  | 3.2 |
| Proquinazid | F | 20.7 | 32.3 |  | 35.0 |  |  |  |  |  |  |
| Pyraclostrobin | F | 120.5 | 90.0 | 3.0 | 156.1 | 11.8 | 10.0 |  | 8.4 | 17.5 | 73.4 |
| Spiroxamine | F | 132.4 | 78.5 | 2.0 | 65.3 | 6.1 | 3.0 |  |  | 27.8 | 174.1 |
| Tebuconazole | F | 7.8 | 23.8 |  | 36.8 |  |  |  |  |  |  |
| Tebufenpyrad | I |  | 6.2 |  |  |  |  |  |  |  |  |
| Thiacloprid | I | 4.2 |  |  |  |  |  |  |  |  |  |
| Trifloxystrobin | F | 6.5 | 4.9 |  | 16.7 |  |  | 2,622.5 |  | 2.1 | 2.2 |

F = Fungicide, I = insecticide, H = herbicide
^a^substances have not been included in this analysis at LUFA Speyer.
^b^unknown plant species: due to problems in the laboratory, not enough pollen was left over for palynological analysis in this sub-fraction.
^c^substances have not been included in this analysis at JKI Berlin.
